# Discovering a novel glycosyltransferase gene *CmUGT1* enhances main metabolites production of *Cordyceps militaris*

**DOI:** 10.3389/fmicb.2024.1437963

**Published:** 2024-10-22

**Authors:** Rong-an He, Chen Huang, Chun-hui Zheng, Jing Wang, Si-Wen Yuan, Bai-Xiong Chen, Kun Feng

**Affiliations:** ^1^School of Bioengineering, Zunyi Medical University, Zhuhai, China; ^2^State Key Laboratory of Quality Research in Chinese Medicine, Macau Institute for Applied Research in Medicine and Health, Macau University of Science and Technology, Macau, China

**Keywords:** *Cordyceps militaris*, DEGs, UDP-glycosyltransferase, gene overexpression, polysaccharides, cordycepin, carotenoids

## Abstract

*Cordyceps militaris* is a filamentous fungus used for both medicinal and culinary purposes. It exhibits a wide range of pharmacological activities due to its valuable contents of cordycepin, polysaccharides, carotenoids, terpenoids and other metabolites. However, *C. militaris* strains are highly susceptible to irreversible degradation in agricultural production, which is often manifested as a prolonged color change period and a significant decrease in the production of secondary metabolites. UDP-glycosyltransferases are an important enzyme family that participates in the synthesis of terpenoids by performing the glycosylation of key residues of enzymes or molecules. However, few studies have focused on its effect on the regulation of metabolite production in *C. militaris*. Therefore, in this study, we performed transcriptome analysis across four different developmental stages of *C. militaris* to target the putative glycosyltransferase gene *CmUGT1,* which plays important roles in metabolite production. We further constructed and screened a *CmUGT1*-overexpressing strain by *Agrobacterium tumefaciens*-mediated infestation of *C. militaris* spores. The major metabolite production of the wild-type and *CmUGT1*-overexpressing *C. militaris* strains was determined after short-term shake-flask cultivation of mycelia. The results showed that the yields of carotenoids and polysaccharides in the mycelia of the *CmUGT1*-overexpressing strains were 3.8 and 3.4 times greater than those in the mycelia of the wild type, respectively (*p* < 0.01). The levels of intracellular and extracellular cordycepin produced by the overexpression strain were 4.4 and 8.0 times greater than those produced by the wild-type strain (*p* < 0.01). This suggests that the overexpression of *CmUGT1* in *C. militaris* enhances the synthesis activities of the main enzymes related to metabolite production, which provides a guide for obtaining excellent recombinant strains of *C. militaris*.

## 1 Introduction

*Cordyceps militaris* is a type of species of the *Cordyceps genus* and a unique edible and medicinal fungus found in Asia (Wang et al., 2023). It contains active ingredients such as cordycepin, polysaccharides, carotenoids, mannitol and therefore exhibits various pharmacological activities (Chen et al., 2021; Wu et al., 2024; Krishna et al., 2023). However, *C. militaris* strains are prone to irreversible degradation in agricultural production (Hoang et al., 2024), usually manifested by vigorous growth of aerial hyphae, a prolonged or no color transition period, and a significant decrease in the production of secondary metabolites (Vu et al., 2023). Although the whole-genome sequence of *C. militaris* CM01 was published in 2011 (Zheng et al., 2011), but most methods used to suppress the degradation of *C. militaris* strains are still focused on farming, and gene editing technology has received less attention.

Carotenoids are a type of tetraterpene with lipid solubility, are a kind of high unsaturated pigments and are important active products of *C. militaris* (Maoka, 2020). It has significant antioxidant, antibacterial, and anticancer activities (Chen P. et al., 2022; Lan et al., 2022; Zhao et al., 2021). According to the structural characteristics of long unsaturated conjugated polyenes, Tang et al. (2019) successfully isolated the significant antioxidant *C. militaris* pigments named cordycepene C_14_H_17_N_1_O_4_ from *C. militaris*. Moreover, gene knockout and *Agrobacterium* infection overexpression experiments demonstrated that the *Cmfhp* gene positively promotes the generation of NO and carotenoids in *C. militaris. Cmfhp* also joined in the regulation of growth and development of *C. militaris* fruiting bodies, which arousing curiosity about the relationship between carotenoids content and fruiting bodies formation in *C. militaris* (Lou et al., 2020). *Cordyceps* polysaccharides are one of the primary active compounds in *C. militaris*. They demonstrate blood sugar-lowering, antioxidant, immune-boosting, antitumor, antibacterial, and anti-inflammatory effects (Miao et al., 2022). The known enzymes involved in the regulation of *Cordyceps* intracellular polysaccharides are glucokinase (GK), UDP glucose pyrophosphate (UGP), phosphoglucose mutase (PGM), and UDP glucose 6-dehydrogenase (UGDH). Besides, Fu et al. found that the residue Arg1436 of β-1,3-glucan synthase (CMGLS), which may play roles in the production of *Cordyceps* polysaccharides, is the key residue directly interacting with the moieties of glucose, phosphate, and UDP glucose (Fu et al., 2022). Wang et al. reported that 4 genes (*gk*, *pgm*, *ugp*, and *upgh*) overexpressed in *C. militaris, respectively,* or combinatorically both preformed an increasing polysaccharide content in *C. militaris* (Wang et al., 2021). Cordycepin is the most famous ingredient in *C. militaris*. It has a similar structure as 2′-deoxyadenosine which lead it to be pharmacological useful, such as antifungal, anti-inflammatory (Tan et al., 2020), antitumor, antiviral, antioxidant (Bibi et al., 2022), and neuroprotective effects (Prommaban et al., 2022). The biosynthesis pathway of cordycepin in *C. militaris* has been reported by Xia et al. (2017). Wongsa et al. proposed alternative pathways for cordycepin biosynthesis by comparing the transcriptome profiles of *C. militaris* cultured in xylose, glucose and sucrose (Wongsa et al., 2020).

UDP-glucosyltransferase (UGT) is one of the most important glycosyltransferases involved in the biosynthesis of disaccharides, oligosaccharides, and polysaccharides (Drula et al., 2022). Recently, researches focused on the interaction between UGT glycosyltransferase and terpenoid compounds production. For example, Wu et al. reported that the UGT glycosyltransferase PpUGT85A2 enhances the aroma and defense ability of peach fruit by controlling the glycosylation of the monoterpenoid linalool (Wu et al., 2019). Xu et al. discovered that the generation of diterpenoid compounds saffrons in saffron because its UDP glycosyltransferase altered the number and position of sugar groups in some compounds of saffron through glycosylation (Xu et al., 2020). Tang et al. reported that the UDP glycosyltransferases PjmUGT1 and PjmUGT2 performed regional glycosylation of oleanolic acid 3-O-β-, and the C-28 carboxyl group and C-3 glucuronic acid group of glucuronic acid, thereby participating in the generation of the triterpenoid compound ginsenoside Ro (Tang et al., 2021). The functions of UGT glycosyltransferase in fungi has been studied. For example, Mao et al. (2023) overexpressed the UDP glycosyltransferase gene *egt* in *Beauveria bassiana*, to deactivate the crucial hormone 20-hydroxyecdysone of silkworm larvae, leading to a significant increase of pathogenicity. Zhang et al. (2024) constructed a UDP glycosyltransferase-expressing strain *PL-UGT* using the endophytic fungus *Phomopsis liquidambaris*. This strain significantly increased the efficiency of converting deoxynivalenol (DON) to DON-3-G *in vitro*, thereby enhancing the plant’s antibacterial ability against *Fusarium graminearum*. However, no research focus on the function of UDP glycosyltransferase in the regulations of valuable metabolites production and fruiting body formation in medicinal fungus.

In this study, we conducted transcriptome sequencing on four samples from different developmental stages of *C. militaris*, namely, CM1 (non-pigmentation stage), CM2 (pigment generation stage), CM3 (primordium stage), and CM4 (fruiting body stage). Through transcriptome sequencing and bioinformatics analysis, a novel UDP glycosyltransferase gene *CmUGT1* was obsearved from differentially expressed genes (DEGs). The gene *CmUGT1* was cloned and overexpressed back into *C. militaris*. After the performing of fermentation and compounds detection of the overexpressed strains, we demonstrated that the UDP glycosyltransferase gene *CmUGT1* can enhance the biosynthesis of carotenoids, polysaccharides, and cordycepin in *C. militaris*. Our results lay a fundation of obtaining high-quality recombinant strains of *C. militaris*.

## 2 Materials and methods

### 2.1 Fungal strain sampling and sequencing

The *C. militaris* strain CM10 was selected for our study. The fruiting bodies were cultured on brown rice media. Four different growth stages of *C. militaris* samples (CM1, CM2, CM3, and CM4) with three replicates in each stage were selected for RNA sequencing. Briefly, the process involved isolating RNA from the fungal sample using the Fungal Total RNA Isolation Kit, followed by preparing a library for deep RNA sequencing. The library preparation protocol was established by Shanghai Sangon Biotech Company following the manufacturer’s standard procedure. This allowed for high-quality RNA sequencing to be conducted on the fungal sample.

### 2.2 Basic bioinformatics analysis

The raw RNA-seq data were assessed by FastQC (v0.11.2; Brown et al., 2017; Mohideen et al., 2020) and leached using Trimmomatic v0.36 (Bush, 2020) with the aim of eliminating those reads and adaptors that were not satisfactory (a base number with a mass value below 15 in a read is greater than 20% and an unknown base N content greater than 5%), and obtain clean reads of the data to be analyzed stored in FASTQ format. The obtained clean data were subsequently combined with the *C. militaris* CM01 genome derived from NCBI via HISAT2 (version: v0.1.2-beta; parameters: --phred64--sensitive--nodiscordant--no-mixed-I1-X1000; Kim et al., 2019). The alignment results were assessed using RSeQC (Vera Alvarez et al., 2019). The duplicate reads were also analyzed using RSeQC. The uniform distribution of reads was tested using Quaimap (Salakhov et al., 2022), and the gene coverage was evaluated using BEDTools (Danecek and McCarthy, 2017; Guo et al., 2020). BCFTools was applied to detect the SNPs and INDELs. Seven different events of alternative splicing were predicted using ASprofile (Florea et al., 2013; Andrews et al., 2021; Li et al., 2020), including exon skipping (SKIP), cassette exons (MSKIP), retention of single (IR), multiple (MIR) introns, alternative exon ends (AE), alternative transcription start site (TSS) and alternative transcription termination site (TTS). Finally, the transcriptome reconstruction on each sample were doing by Stringtie software (version: v1.0.4; parameters: -f0.3 -j3 -c 5 -g100 -s 10000 -p8). Then use Cuffcompare (Trapnell et al., 2012; version: v2.2.1; Parameters: -p12) to compare the reconstructed transcript with the reference annotation information to obtain new transcripts and isoforms.

### 2.3 Differential expression analysis

The expression level of each transcript was calculated using the TPM (transcripts per million) calculated by StringTie as follows:


$$TPM_{i}=\frac{X_{i}}{L_{i}}*\frac{1}{\sum_{j}\frac{X_{j}}{L_{j}}}*10^{6}$$

X_i_ = total exon fragment/reads.

L_i_ = exon length/KB.

The R package DESeq (Chen B. Y. et al., 2022), which is suitable for R statistical computing, was used to analyze the differentially expressed transcripts. The three replicates of four different growth stages were compared. We used the Benjamini–Hochberg method for multiple test *p* values for differential expression analysis and retained only the transcripts with a false discovery rate (FDR) ≤0.05 and a fold change| > =2. To survey the functions of the differentially expressed transcripts, we performed a functional enrichment analysis of the groups of differentially expressed transcripts via ClueGo in Cytoscape.

### 2.4 Phylogenetic analysis

Alignments were performed with the ClustalW method, and phylogenetic trees were generated using the neighbor-joining method and p-distance model (bootstrap resampling of 1,000 replicates) through MEGA 11.0 (Gao et al., 2024). The data subsets of gaps and/or missing data were treated as complete deletions. The *CCM-09558* (renamed as *CmUGT1* in this research) protein sequences of other species were used for tree construction together with the sequences generated in this study.

### 2.5 Construction of the p390-BlpR-CmUGT1 overexpression vector

A template suspension of *C. militaris* mycelium was obtained by grinding with Buffer (100 mM Tris–HCl pH 9.5, 1 M KCl, and 10 mM EDTA). The PCR amplification of target genes (primers 5′-TAGGATCCGGCTTGCCTATGAGCAAACC-3′ and 5′-CTGTTCCTATTCTTGCCGAAGTCA-TGGCTAACTGAAGGCG-3′) was performed using 2× Phanata Polymerase DNA (Vazyme Biotech Co., Ltd.). The purified DNA sequence of the glycosyltransferase gene *CmUGT1* was subsequently inserted into our previous reported plasmid p390-BlpR-Pcmtf1(Chen, B. X. et al., 2022) using a 2 × seamless cloning kit. The recombinant overexpression shuttle plasmid p390-BlpR-CmUGT1 was transformed into *A. tumefaciens* AGL-1 for the construction of *C. militaris* transformants.

### 2.6 Construction of *CmUGT1*-overexpressing transformants of *C. militaris*

As previous, 1–2 ml of 0.05% Tween 80 solution was added to the surface of CM10 mycelium of *C. militaris* cultured in the dark for 14–21 days and exposed to light for 5–7 days (Chen, B. X. et al., 2022). Then, the *C. militaris* spores was collected through filtration. The resuspension of *C. militaris* spores was mixed with *Agrobacterium tumefaciens* carrying shuttle carriers at ratios of 1:19, 1:9, and 1:5 in regeneration media and incubated at 25°C in a dark environment for 2–3 days. When white spots (with a diameter of 1 mm) sprouted on the coculture plate, they were transferred to M-100 selective media (containing 300 μg/ml cephalosporin and 600 μg/ml phosphinothricin). Overexpressed transformants were incubated in a dark environment at 25°C for 5–7 days. Multiple plate screenings were conducted, and biological samples (*n* > 3) were cultured for the *CmUGT1*-overexpression strain. Each sample underwent more than three technical replicates (Supplementary Figure S10; Supplementary Tables S9–S12), which is similar to previous research (Lou et al., 2020). The mycelium growth status of overexpression transformants on PDA plate were compared with wild-type in a period of 3-days darkness plus 3-days light. The fermentation of the *CmUGT1-*overexpression and the wild-type strain of *C. militaris* were performed in PPSB medium at 25°C with 220 rpm for 8 days (3-days darkness plus 5-days light).

### 2.7 Detection of the main metabolites in *C. militaris*

The overexpressed strain of *C. militaris* fermented for 8 days (dark culture for 3 days and light exposure for 5 days) was centrifuged at 12,000 rpm and 4°C for 20 min. The supernatant were filtered for UPLC detecting. The mycelia collected by centrifugation was dried overnight in a 56°C oven, 20 mg of dried mycelia were accurately weighed and immersed in 2 ml of 20% methanol. The tube was then sonicated in a water bath using an ultrasonic instrument for 1 h. After thorough vortexing following the ultrasonication, the sample was centrifuged at 15,500 rpm for 30 min under 25°C, and the supernatant was filtered for UPLC analysis (Liu et al., 2019). Using a Model LC2000 liquid chromatograph, a Welch Ultimate AQ-C18 UPLC column (4.6 × 250 mm, 5 μm) was used. The mobile phase consisted of 85% ultrapure water and 15% (v/v) methanol (Lin et al., 2020), with a flow rate of 2 ml/min, a column temperature of 40°C, and a detection wavelength of 260 nm. The sample was injected with 2 μl each time. A concentration standard curve was generated using the peak area normalization method.

The content of carotenoids in mycelia are extracted by the acid heating method (Lou et al., 2020; Chen P. et al., 2022). In brief, the wild-type and the *CmUGT1-*overexpression strain were fermented for 8 days (dark culture for 3 days and light exposure for 5 days). The mycelia and supernatant were separated by centrifugation, the mycelia were collected and dried at 56°C. 20 mg of dry samlpes were weighed and scanned using UV spectrophotometry. The maximum absorption wavelength of 445 nm was selected for carotenoid quantification.

The carotenoid content (μg/g) was calculated based on the following equation:

Carotenoid content (μg/g) = (A × V × D)/(0.16 × W), where A is the absorbance value of the diluted extraction at λ_max_, V is the volume of the extraction reagent, D is the dilution ratio, 0.16 is the extinction coefficient of carotenoids, and W is the weight (g) of the dry mycelia of *C. militaris.*

Fermentation of wild-type and the *CmUGT1*-overexpression *C. militaris* strains were performed for 8 days (dark culture for 3 days, light exposure for 5 days), followed by centrifugation separation of the mycelia and supernatant, and determination of the *C. militaris* polysaccharide content using the supernatant according to a previously reported phenol sulfuric acid method (He et al., 2019).

### 2.8 Statistical analysis

All data presented were repeated three times, and statistical analysis of data was conducted using SPSS 22.0, with values expressed as mean ± standard error. A *p*-value less than 0.05 is considered significant.

## 3 Results

### 3.1 Basic data processing, quality assessment, alignment, and assembly

**Figure 1:**
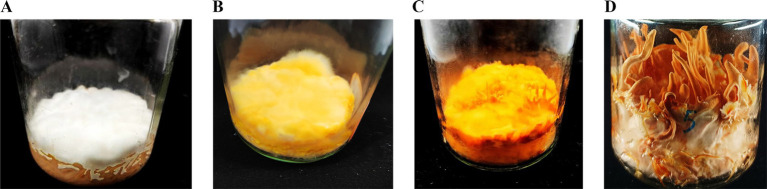
The growth morphology of *C. militaris* CM10 in brown rice medium under alternating light conditions. **(A)** CM1, cultivated in the dark for 7 days. **(B)** CM2, cultivated in the dark for 7 days, followed by light exposure for 7 days. **(C)** CM3, cultivated in the dark for 7 days, followed by light exposure for 15 days. **(D)** CM4, cultivated in the dark for 7 days, followed by light exposure for 35 days.

Four different growth stages of the *C. militaris* samples (CM1, CM2, CM3, CM4) were shown in Figure 1. All the RNA samples were sequenced on an Illumina HiSeq 3,000 platform, which yielded an average of 62,425,693 reads for each sample. All the raw data were subjected to FastQC (Luo et al., 2023) for sequencing quality assessment and were leached to remove unsatisfactory reads and adapters using Trimmomatic (van Heusden et al., 2022; Supplementary Table S1). The obtained clean reads were aligned against *C. militaris* genome (CM01_v01) using HISAT2 (Kim et al., 2019). This results illustrated that the vast majority of the clean reads (more than 90%) could be aligned to the genome sequences of *C. militaris* (Supplementary Table S2). In addition, analysis of the PCR duplication rate and uniform distribution was conducted using RSeQC (Vera Alvarez et al., 2019) and Qualimap (Salakhov et al., 2022), respectively, which indicated that the quality of these mapped reads was good enough for further study (Supplementary Figures S1, S2). Afterwards, the alignment results (bam files) were subjected to StringTie (Pronozin and Afonnikov, 2023) for transcript assembly. Apart from 9,651 known transcripts that have already been detected in the reference genome CM01, 10,898 novel transcripts were identified in our study. The discovery of these new transcripts provides extended possibility for the subsequent exploration of valuable functional genes.

### 3.2 Variants and alternative splicing analysis

**Figure 2:**
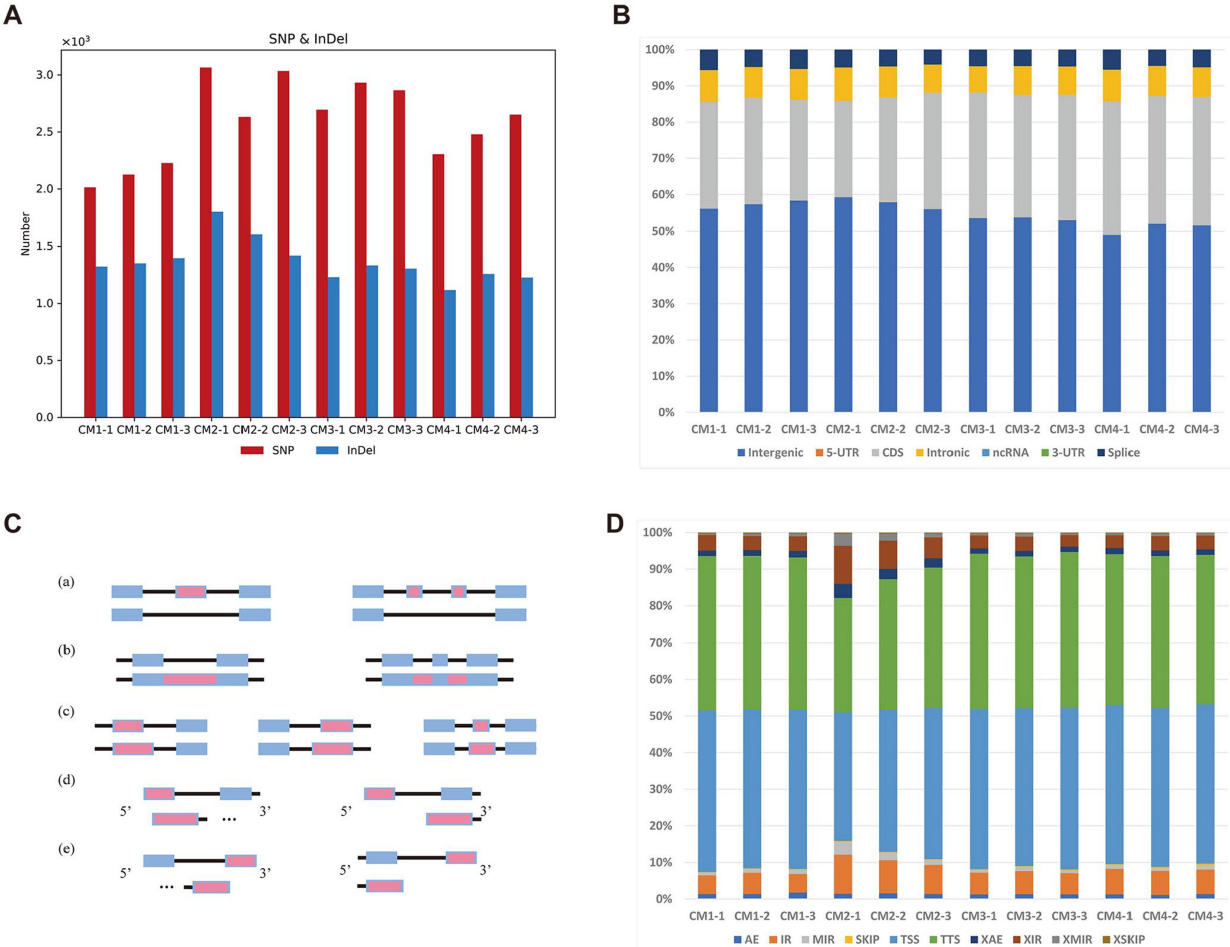
Variants and alternative splicing analysis of different developmental stages of *C. militaris.* **(A)** Each bar represents the total number of mutations (SNPs and INDELs) in four stages. **(B)** Each bar represents the faction of mutations (SNPs and INDELs) annotated by SnpEff in four stages. **(C)** Five alternative splicing (AS) modes identified in stages of *C. militaris*, including (a) Exon skipping (SKIP) and cassette exons (MSKIP); (b) retention of single (IR) and multiple(MIR) introns; (c) alternative exon ends (AE); (d) alternative transcription start site (TSS); (e) alternative transcription termination site (TTS). Alternatively spliced feature are shown in red. **(D)** Each bar represents the faction of AS modes identified in four stages.

To determine whether the current alternative splicing RNA exhibits different manifestations in different developmental stages of *C. militaris*. For SNP/INDEL calling, this study used BCFTools (Danecek et al., 2021) to detect those variants for each sample. A total of 15,389 SNPs/INDELs were detected among those samples (Figure 2A). Basic annotation for these variants achieved by SnpEff (Colomer-Vilaplana et al., 2022) indicated that there was no difference in these variants across these four different growth stages in *C. militaris* transcriptome (Figure 2B). On the other hand, we also investigated seven different AS (alternative splicing) events that occurred among the four growth stages in *C. militaris* (Figure 2C) using the ASprofile (Lorenzo et al., 2020). The results showed that TSSs (alternative transcription start sites) and TTSs (alternative transcription termination sites) accounted for most of the AS events in the *C. militaris* transcriptome, but no AS events were clearly present in a distinct proportion of these different growth stages (Figure 2D). We conducted a characteristic analysis of alternative splicing RNAs in samples of *C. militaris* at different developmental stages. And found that the proportions of these alternative splicing RNAs did not show significant differences, which were primarily presented at the alternative splicing of transcription start sites (TSS) and transcription termination sites (TTS). This suggests that the knockout and overexpression of certain genes are particularly crucial for the normal growth and development of *C. militaris*, providing strategic directions for the study of gene expression polymorphism and the diversity of protein functions.

### 3.3 Cluster analysis of DEGs revealed distinct expression patterns in four growth stages of *C. militaris*

According to our transcriptome sequencing analysis, the most significantly enriched genes in CM2/CM1 were related to secondary metabolic process (log_2_Fold = 4.17, *p* = 0.00074), extracellular region (log_2_Fold = 4.05, *p* = 2.3 × 10^−13^), and oxidoreductase activity (log_2_Fold = 6.54, *p* = 0.000054; Supplementary Figure S3; Supplementary Table S3). Among CM3/CM2, the most significantly enriched terms were carbohydrate metabolic process (log_2_Fold = 2.87, *p* = 0.000053), integral component of plasma membrane (log_2_Fold = 1.96, *p* = 0.00038), and inorganic phosphate transmembrane transporter activity (log_2_Fold = 2.63, *p* = 0.00013; Supplementary Figure S4; Supplementary Table S4). In CM4/CM3, the most significantly enriched terms were lipid biosynthetic process (log_2_Fold = 5.34, *p* = 0.0000001), oxidoreductase complex (log_2_Fold = 3.22, *p* = 0.0000073), and transporter activities (log_2_Fold = 2.42, *p* = 0.00037; Supplementary Figure S5; Supplementary Table S5). GO data analysis indicated that DEGs involved in metabolic processes, organelles, and catalytic activity may be directly involved in the growth and development of *C. militaris*. The most significant enrichment of DEGs in CM2/CM1 occurred through galactose metabolism (log_2_Fold = 5.94, *p* = 0.013997758), cyanoamino acid metabolism (log_2_Fold = 5.55, *p* = 0.006050669) and fatty acid degradation (log_2_Fold = 5.31, *p* = 0.000736841; Supplementary Figure S6; Supplementary Table S6). This suggests that the development of *C. militaris* non-pigmentation into pigment generation may require a large amount of energy, which is potentially related to glycolysis metabolism in the carbon center pathway. The most significant enrichment of DEGs in CM3/CM2 occurred through the pathways of glycerophospholipid metabolism (log_2_Fold = 2.03, *p* = 0.003834), ether lipid metabolism (log_2_Fold = 2.03, *p* = 0.004261) and quorum sensing (log_2_Fold = 2.03, *p* = 0.006165; Supplementary Figure S7; Supplementary Table S7). This indicates that in the pigment generation and primordium, the DEGs were enriched in the pathways of galactose metabolism, starch and sucrose metabolism, indicating that glycosyltransferases may transfer one glycosyl molecule to another receptor during this process, regulating the rate and direction of sugar metabolism to promote the development of *C. militaris*. The most significant enrichment of DEGs in CM4/CM3 fruiting bodies occurred through fatty acid metabolism (log_2_Fold = 4.63, *p* = 0.00023), amino sugar and nucleotide sugar metabolism (log_2_Fold = 3.79, *p* = 0.000413), carbon metabolism (log_2_Fold = 3.67, *p* = 0.000445; Supplementary Figure S8; Supplementary Table S8), indicating that most of the DEGs were involved in energy synthesis, once again suggesting the important role of carbon center metabolism in the growth and development of *C. militaris*. Interestingly, when screening genes through differential multiples, GO, and KEGG differential enrichment pathways, multiple UDP glycosyltransferase genes were found to be significantly differentially expressed during CM2/CM1 but did not appear in the KEGG differential enrichment pathway (Table 1). The GO and KEGG enrichment data across each developmental stages of *C. militaris*, showed that there are varying pattern of metabolism during different stages. Among the significant enrichment pathways, the pathway which contains the UDP glycosyltransferase genes is particularly prominent, providing potential targets for performing gene functions validation by wet experiments.

**Table 1:** The transcript level of UDP glycosyltransferase DEGs in *C. militaris* during developmental stages.

Gene ID	Expression (CM1)	Expression (CM2)	Expression (CM3)	Expression (CM4)
CCM_09558	4.72	511.06	368.61	299.63
CCM_01719	31.60	6.81	1.56	6.34
CCM_02376	32.49	19.41	20.20	24.83
CCM_09110	13.52	21.56	21.72	16.99
CCM_01158	42.95	13.39	24.64	55.63
CCM_03311	36.40	12.38	22.41	45.44
CCM_05430	123.36	51.68	82.33	125.88
CCM_07583	105.38	38.62	41.08	92.83
CCM_03951	15.93	21.82	20.92	9.97
CCM_03309	134.03	8.54	7.05	8.48
CCM_09129	9.70	32.96	22.63	8.19
CCM_01415	4.97	2.97	1.09	0.31
CCM_01686	3.46	4.71	3.02	7.06
CCM_04175	42.31	27.67	36.79	45.68
CCM_01179	17.02	10.90	7.69	6.42
CCM_02123	16.58	13.53	12.11	7.21
CCM_02414	53.83	21.71	23.53	30.41

### 3.4 Construction and screening of *CmUGT1*-overexpressing transformants of *C. militaris*

**Figure 3:**
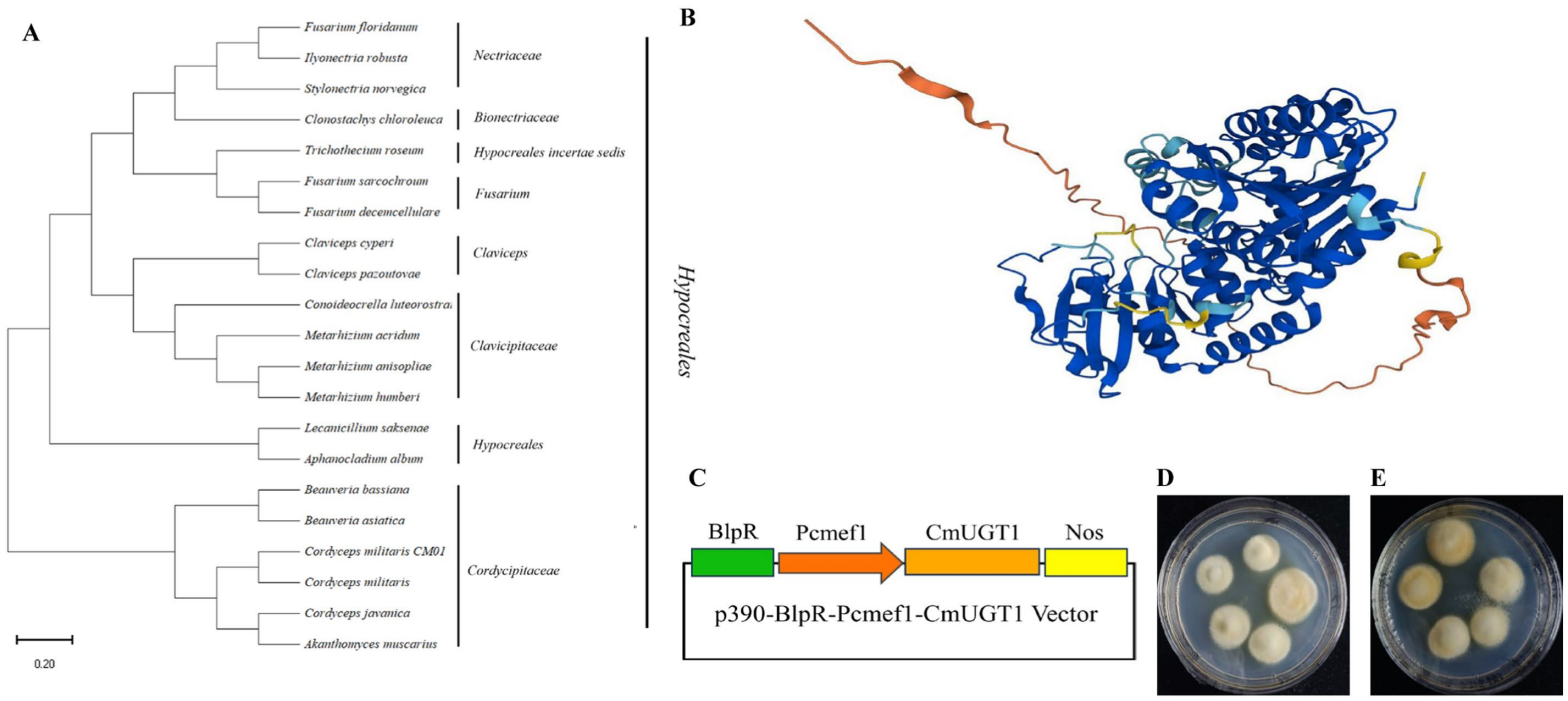
Construction of the recombinant plasmid and screening of transformants. **(A)** Phylogenetic trees of *CmUGT1* from filamentous fungi. The topology of these trees was generated using MEGA 11.0. The scale bar represents 0.2 substitutions per site for *CmUGT1*. **(B)** 3D structural diagram of *CmUGT1* predicted by Alphafold 1.0 from NCBI. **(C)** Schematic diagram of recombinant plasmid construction. The green square represents the resistance gene, the orange square arrow indicates the *Pcmef1* promoter, the brown square represents the target gene *CmUGT1*, the yellow square represents the terminator. **(D)** Morphological diagram of the wild-type mycelium of *C. militaris* CM10. **(E)** Morphological diagram of the mycelium of *C. militaris* CM10 with overexpressing of *CmUGT1* gene.

Due to the uniqueness of the carotenoid biosynthesis pathway and the fact that previous research on UDP glycosyltransferases has focused mostly on the biosynthesis of terpenoids, this gene was not reported in the carotenoid biosynthesis pathway map00906. Therefore, we chose this gene as our research object. A database search conducted through GenBank NCBI BLASTN showed that the predicted gene *CmUGT1* was highly similar to the UDP-glycosyltransferase (*Beauveria bassiana*). A phylogenetic tree was constructed to retrieve the amino acid sequences of relevant species from the NCBI database. The constructed evolutionary tree showed that the species in which this amino acid is present all belong to the *Ascomycota* family, indicating that this amino acid has strong specificity, and a subbranch has been formed in these *Ascomycota* species (Figure 3A). In addition, a three-dimensional structural prediction analysis of the amino acids was also conducted. It is a transmembrane hydrophilic protein that contains 519 amino acids and has a molecular weight of 56,069 Da. The three-dimensional structure shows that there is no homologous protein that is 100% similar to it, indicating a specific hydrophilic protein structure (Figure 3B). Recombinant plasmid construction was verified by colony PCR and enzyme digestion, indicating successful construction of the recombinant plasmid p390-BlpR-Pcmef1-CmUGT1 (Figure 3C; Supplementary Figures S9A,B). The identification of the overexpressed strains of *C. militaris* is shown in Supplementary Figures S10A,B. Importantly, the overexpression of the transformants was more pronounced than that of the wild type in terms of color transformation in *C. militaris* (Figures 3D,E). The homologous protein from *Beauveria bassiana* was expressed heterologously in *Escherichia coli* to verify its ability to catalyze the glycosylation of flavonoids to form flavonoid glucosides *in vitro*; however, it remains uncertain whether the homologous *CmUGT1* enzyme in *C. militaris* may have different functions, and whether it affects the levels of the main metabolites in *C. militaris* has yet to be determined. Therefore, this study aims to construct overexpressing strains to further validate its function (Matera et al., 2024). Additionally, a strain capable of overexpressing the UDP glycosyltransferase gene *CmUGT1* was successfully constructed through *Agrobacterium* infection, providing a foundation for further investigation into the biochemical indicators associated with this gene in *C. militaris*.

### 3.5 Detection of the main metabolites of *CmUGT1*-overexpressing transformants of *C. militaris*

**Figure 4:**
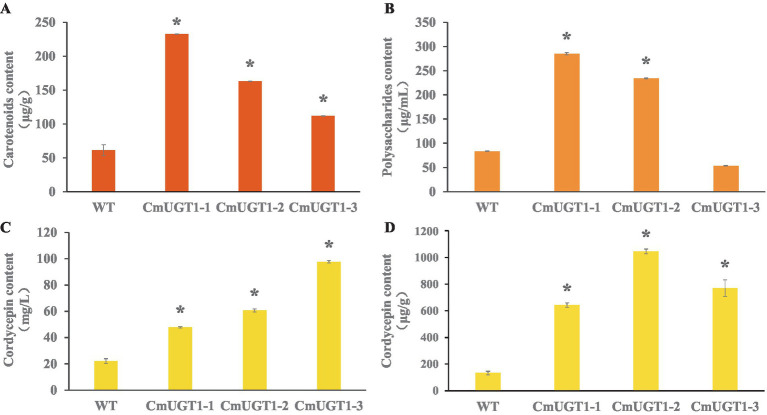
Detection of main metabolites content of *C. militaris* fermentative sampls between wild type and three replicates of the *CmUGT* overexpressed transformants. **(A)** The carotenoid content of fermentatived mycelium. **(B)** The polysaccharides content of fermentatived broth. **(C)** The cordycepin content in fermentatived broth. **(D)** The cordycepin content of fermentatived mycelium. WT indicates wild-type strains, CmUGT1-1 ~ CmUGT1-3 indicates overexpressed strains that were randomly picked from transformants, all statistical analyses were conducted on the WT, *n* = 3, “*” indicates *p* < 0.01.

The contents of the main metabolites carotenoids, cordycepin and polysaccharides in the *C. militaris* strain overexpressing the *CmUGT1* gene were significantly greater than those in the wild-type strain. *CmUGT1*-1, *CmUGT1*-2, and *CmUGT1*-3 are three overexpressed transformation factors that were randomly inserted. The content of carotenoids in strain overexpressing *CmUGT1* was 3.8 times (*p* < 0.01) greater than that in wild-type strain (Figure 4A; Supplementary Table S9), indicating that the *CmUGT1* gene can promote the biosynthesis of pigments in *C. militaris*. The polysaccharide content of *CmUGT1*-overexpressing strain was 3.4 times (*p* < 0.01) greater than that of wild-type strain (Figure 4B; Supplementary Table S10), indicating that the *CmUGT1* gene can promote the biosynthesis of polysaccharides in *C. militaris*. The content of cordycepin in strain overexpressing *CmUGT1* was 4.4 times (*p* < 0.01) greater than that in wild-type strain (Figure 4C; Supplementary Figures S11B,C; Supplementary Table S11), and the mycelium content was 8.0 times (*p* < 0.01) greater than that in wild-type strain (Figure 4D; Supplementary Figures S12B,C; Supplementary Table S12), indicating that the *CmUGT1* gene can promote the biosynthesis of cordycepin in *C. militaris*. By fermentation and detection of the *CmUGT1* overexpressing strain of *C. militaris*, the results indicate that the yields of carotenoids, polysaccharides, and cordycepin in the *CmUGT1* overexpressing strain are significantly higher than those in the wild-type strain, with significant differences observed. These results demonstrated that enhancing the *in vivo* expression of *CmUGT1* would increase the production of the main metabolites of *C. militaris*.

## 4 Discussion

*C. militaris* has become an important health and medicinal material since it harbors a variety of secondary metabolites that are good for the human body, i.e., water-soluble carotenoids (Shevchuk et al., 2023). However, its peculiar lifestyle covers one layer of mysterious veil on the induction of the primordium, sexual reproduction and the molecular basis of its lifecycle. Systematic transcriptome analysis of its developmental process will help us to understand the basic biology of this fungus and benefit its large-scale cultivation. In the present study, we not only compared the transcriptomes of four different developmental stages of *C. militaris* at the transcriptional level but also compared them at the structural level. For the comparison of structural levels, we initially detected the SNPs and INDELs as well as their possible ASs among the different development-stage samples. The results showed that there was no significant difference in the variants exhibited in these samples. It is rational that different development stages should not introduce mutations that undoubtedly affect the growth and development of *C. militaris*. With the progress of transcriptome sequencing of carotenoid metabolism flux in *C. militaris* (Soommat et al., 2024), this topic has become increasingly popular. The content of carotenoids in *C. militaris* has been recommended as one of the quality standards for measuring its commercial value.

Currently, there is little genetic research on the carotenoids in *C. militaris* and even less research on the relationship between *C. militaris* carotenoids and UDP glycosyltransferase. Previous studies on UDP glycosyltransferase revealed that it can promote the glycosylation of terpenoids and change the position and quantity of glycosides (Yuan et al., 2024), thereby promoting the synthesis of terpenoids and exhibiting different epigenetic phenomena in different hosts. Lou et al. demonstrated through gene supplementation experiments that the *Cmfhp* gene of flavin hemoglobin may promote the production of carotenoids by reducing NO content, ultimately restoring the normal traits of defective *C. militaris* (Lou et al., 2020), Furthermore, transcriptome analysis and the same experimental approach were used to demonstrate that the *Cmtns* gene can significantly promote carotenoid biosynthesis in *C. militaris*, thereby elucidating the carotenoid biosynthesis pathway in this species (Lou et al., 2019). In this study, the *CmUGT1* strain overexpressing the UDP glycosyltransferase gene produced 3.8 times more carotenoids than did the wild-type strain, which is not a carotenoid synthesis pathway gene in *C. militaris*. This once again proves the uniqueness of carotenoid synthesis in *C. militaris* and may reveal a new biosynthetic pathway, providing a reference basis for revealing the synthesis of carotenoids in *C. militaris*.

Polysaccharides have also received much attention due to their various active functions, such as anti-inflammatory, antibacterial, and antitumor effects (Miao et al., 2022). Currently, they can be used to increase polysaccharide production by overexpressing key genes involved in polysaccharide biosynthesis, co-overexpressing genes, and blocking polysaccharide synthesis metabolic pathways. For example, in a study of *C. militaris* polysaccharides by Xu, the polysaccharide content produced by co-overexpressing CM-Kre5-CMT1-A16 through *Agrobacterium* infection was significantly greater than that produced by single gene expression (Xu et al., 2023). Wang et al. (2021) overexpressed four genes (*gk*, *pgm*, *ugp*, and *upgh*) in the fungus *C. militaris* and found that the polysaccharide content produced by the *C. militaris* mutant strain overexpressing the *pgm* gene was 1.34 times higher than that of the wild-type strain. In addition, the combined overexpression of the *pgm* and *upgh* genes in the engineered *C. militaris* strain increased the polysaccharide production to 1.78 times that of the wild-type strain. In this study, the *CmUGT1* strain overexpressing the UDP glycosyltransferase gene produced 3.4 times more polysaccharides than did the wild-type strain, but its possible metabolic mechanism needs further research.

Cordycepin is an adenosine analog with extensive anti-inflammatory, antibacterial, and antitumor pharmacological activities due to its ability to inhibit RNA synthesis. However, the biosynthetic pathway of cordycepin in *C. militaris* is still divergent. In the early stages, adenine and ribose polymerize through glycosidic bonds to produce cordycepin (Kredich and Guarino, 1961), making it a precursor to cordycepin synthesis. After 1976, it was discovered that the biosynthesis of cordycepin in *C. militaris* is based on adenosine as the precursor, which is ultimately converted into the nucleotide metabolism intermediate 3′-deoxyadenosine through the action of various enzymes, such as adenylate kinase (ADEK), 5′-nucleotidase (NT5E; Xiang et al., 2014), metal-dependent hydrolase CNS2, oxidoreductase/dehydrogenase CNS1 (Xia et al., 2017), etc. With the continuous clarification of the biosynthetic pathways of *C. militaris*, gene editing technology is increasingly being widely applied in the study of cordycepin biosynthesis in *C. militaris*. Yang et al. found that gene supplementation experiments demonstrated that the blue light receptor *CmWC-1* promotes the biosynthesis of cordycepin in *C. militaris*. The content of cordycepin in the supplementation strain was 2.5 times greater than that in the knockout strain (Yang et al., 2016). Zhang et al. found through overexpression of RNRs genes in *C. militaris* that the cordycepin of RNRM overexpressing strains is 1.5 times greater than that of wild-type strains, possibly through hydrolysis of adenosine (Zhang et al., 2020). Chen et al. (2020) reported that overexpression of transcription factors *CmTf1* and *CmTf2* in *C. militaris* resulted in a more than 1-fold increase in cordycepin content. In this study, the *CmUGT1* strain overexpressing the UDP glycosyltransferase gene produced 4.4-fold and 8.0-fold more extracellular and intracellular cordycepin than did the wild-type strain. A significant difference from previous studies is that these genes are relatively rare in the study of cordycepin in *C. militaris*. Additionally, the increase in cordycepin content is also more significant. *C. militaris* may provide more energy to activate the tricarboxylic acid cycle through the glycolytic pathway of carbon metabolism, providing a basis for further improvement of cordycepin in *C. militaris*.

## 5 Conclusion

In this study, a UDP glycosyltransferase gene, *CmUGT1*, was identified from the transcriptome data of different developmental stages of *C. militaris*. The positive function of *CmUGT1* for breeding better traits of *C. militaris* was validated by constructing a *CmUGT1*-overexpressing *C. militaris* transformants to perform metabolites fermentation. The metabolites detection showed that the overexpressing of *CmUGT1* would significantly increase the yield of major bioactive compounds in *C. militaris*. This not only provides a new target for obtaining strains of *C. militaris* with superior characteristics for agriculture and pharmaceutical production, but also points out a new direction for co-expressing other enzyme genes to further improve the content of main metabolites in *C. militaris*. Additionally, it offers new opportunities for future drug development and medical applications.

## Data Availability

The datasets presented in this study can be found in online repositories. The names of the repository/repositories and accession number(s) can be found below: https://www.ncbi.nlm.nih.gov/, PRJNA592219.

## References

[ref1] Andrews T. S. Kiselev V. Y. McCarthy D. Hemberg M. (2021). Tutorial: guidelines for the computational analysis of single-cell RNA sequencing data. Nat. Protoc. 16, 1–9. doi: 10.1038/s41596-020-00409-w, PMID: 33288955

[ref2] Bibi S. Hasan M. M. Wang Y. B. Papadakos S. P. Yu H. (2022). Cordycepin as a promising inhibitor of SARS-CoV-2 RNA dependent RNA polymerase (RdRp). Curr. Med. Chem. 29, 152–162. doi: 10.2174/0929867328666210820114025, PMID: 34420502

[ref3] Brown J. Pirrung M. McCue L. A. (2017). FQC dashboard: integrates FastQC results into a web-based, interactive, and extensible FASTQ quality control tool. Bioinformatics 33, 3137–3139. doi: 10.1093/bioinformatics/btx373, PMID: 28605449 PMC5870778

[ref4] Bush S. J. (2020). Read trimming has minimal effect on bacterial SNP-calling accuracy. Microb. Genom. 6, 1–34. doi: 10.1099/mgen.0.000434, PMID: 33332257 PMC8116680

[ref5] Chen B. X. Xue L. N. Wei T. Ye Z. W. Li X. H. Guo L. Q.. (2022). Enhancement of ergothioneine production by discovering and regulating its metabolic pathway in Cordyceps militaris. Microb Cell Fact. 21, 169–182. doi: 10.1186/s12934-022-01891-5 35999536 PMC9396837

[ref6] Chen P. Gao G. Xu Y. Jia P. Li Y. Li Y.. (2022). Novel gene signature reveals prognostic model in acute lymphoblastic leukemia. Front. Cell Dev. Biol. 10:1036312. doi: 10.3389/fcell.2022.1036312, PMID: 36407095 PMC9669305

[ref7] Chen B. Y. Huang H. S. Tsai K. J. Wu J. L. Chang Y. T. Chang M. C.. (2022). Protective effect of a water-soluble carotenoid-rich extract of *Cordyceps militaris* against light-evoked functional vision deterioration in mice. Nutrients 14, 1675–1687. doi: 10.3390/nu14081675, PMID: 35458237 PMC9031935

[ref8] Chen B. X. Wei T. Xue L. N. Zheng Q. W. Ye Z. W. Zou Y.. (2020). Transcriptome analysis reveals the flexibility of cordycepin network in *Cordyceps militaris* activated by L-alanine addition. Front. Microbiol. 11, 577–593. doi: 10.3389/fmicb.2020.00577, PMID: 32390960 PMC7193312

[ref9] Chen Y. Wu Y. Li S. Du S. Hao X. Zhang J.. (2021). Large-scale isolation and antitumor mechanism evaluation of compounds from the traditional chinese medicine *Cordyceps militaris*. Eur. J. Med. Chem. 212, 113–142. doi: 10.1016/j.ejmech.2020.113142, PMID: 33450619

[ref10] Colomer-Vilaplana A. Murga-Moreno J. Canalda-Baltrons A. Inserte C. Soto D. Coronado-Zamora M.. (2022). PopHumanVar: an interactive application for the functional characterization and prioritization of adaptive genomic variants in humans. Nucleic Acids Res. 50, D1069–D1076. doi: 10.1093/nar/gkab925, PMID: 34664660 PMC8728255

[ref11] Danecek P. Bonfield J. K. Liddle J. Marshall J. Ohan V. Pollard M. O.. (2021). Twelve years of SAMtools and BCFtools. Gigascience 10, 1–4. doi: 10.1093/gigascience/giab008, PMID: 33590861 PMC7931819

[ref12] Danecek P. McCarthy S. A. (2017). BCFtools/csq: haplotype-aware variant consequences. Bioinformatics 33, 2037–2039. doi: 10.1093/bioinformatics/btx100, PMID: 28205675 PMC5870570

[ref13] Drula E. Garron M. L. Dogan S. Lombard V. Henrissat B. Terrapon N. (2022). The carbohydrate-active enzyme database: functions and literature. Nucleic Acids Res. 50, D571–D577. doi: 10.1093/nar/gkab1045, PMID: 34850161 PMC8728194

[ref14] Florea L. Song L. Salzberg S. L. (2013). Thousands of exon skipping events differentiate among splicing patterns in sixteen human tissues. F1000Res. 2, 188–212. doi: 10.12688/f1000research.2-188.v2, PMID: 24555089 PMC3892928

[ref15] Fu X. Zan X. Y. Sun L. Tan M. Cui F. J. Liang Y. Y.. (2022). Functional characterization and structural basis of the β-1,3-glucan synthase cmgls from mushroom *Cordyceps militaris*. J. Agric. Food Chem. 70, 8725–8737. doi: 10.1021/acs.jafc.2c03410, PMID: 35816703

[ref16] Gao X. Gao Z. Zhang M. Qiao H. Jiang S. Zhang W.. (2024). Identifying relationships between glutathione s-transferase-2 single nucleotide polymorphisms and hypoxia tolerance and growth traits in *macrobrachium nipponense*. Animals (Basel) 14, 666–679. doi: 10.3390/ani14050666, PMID: 38473051 PMC10930884

[ref17] Guo G. Chen A. Ye L. Wang H. Chen Z. Yan K.. (2020). TCONS_00483150 as a novel diagnostic biomarker of systemic lupus erythematosus. Epigenomics 12, 973–988. doi: 10.2217/epi-2019-0265, PMID: 32677847

[ref58] He B. L. Guo L. Q. Zheng Q. W. Lin S. X. Lin J. F. Wei T.. (2019). A simple and effective method using macroporous resins for the simultaneous decoloration and deproteinisation of Cordyceps militaris polysaccharides. Int. J. Food Sci. Technol. 54, 1741–1751. doi: 10.1111/ijfs.14063

[ref18] Hoang C. Q. Duong G. H. T. Tran M. H. Vu T. X. Tran T. B. Pham H. T. N. (2024). Molecular mechanisms underlying phenotypic degeneration in *Cordyceps militaris*: insights from transcriptome reanalysis and osmotic stress studies. Sci. Rep. 14, 2231–2243. doi: 10.1038/s41598-024-51946-3, PMID: 38278834 PMC10817986

[ref19] Kim D. Paggi J. M. Park C. Bennett C. Salzberg S. L. (2019). Graph-based genome alignment and genotyping with HISAT2 and HISAT-genotype. Nat. Biotechnol. 37, 907–915. doi: 10.1038/s41587-019-0201-4, PMID: 31375807 PMC7605509

[ref20] Kredich N. M. Guarino A. J. (1961). Studies on the biosynthesis of cordycepin. Biochim. Biophys. Acta 47, 529–534. doi: 10.1016/0006-3002(61)90546-7, PMID: 13754189

[ref21] Krishna K. V. Ulhas R. S. Malaviya A. (2023). Bioactive compounds from *Cordyceps* and their therapeutic potential. Crit. Rev. Biotechnol. 44, 753–773. doi: 10.1080/07388551.2023.2231139, PMID: 37518188

[ref22] Lan L. Wang S. Duan S. Zhou X. Li Y. (2022). *Cordyceps militaris* carotenoids protect human retinal endothelial cells against the oxidative injury and apoptosis resulting from H_2_O_2_. Evid. Based Complement. Alternat. Med. 2022, 1259093–1259105. doi: 10.1155/2022/1259093, PMID: 36212977 PMC9546680

[ref23] Li Y. H. Zou M. Han Q. Deng L. R. Weinshilboum R. M. (2020). Therapeutic potential of triterpenoid saponin anemoside B_4_ from Pulsatilla chinensis. Pharmacol. Res. 160, 105079–105137. doi: 10.1016/j.phrs.2020.105079, PMID: 32679180

[ref24] Lin L. Z. Wei T. Yin L. Zou Y. Bai W. F. Ye Z. W.. (2020). An efficient strategy for enhancement of bioactive compounds in the fruit body of caterpillar medicinal mushroom, *Cordyceps militaris* (*Ascomycetes*), by spraying biotic elicitors. Int. J. Med. Mushrooms 22, 1161–1170. doi: 10.1615/IntJMedMushrooms.2020037155, PMID: 33463933

[ref25] Liu X. C. Zhu Z. Y. Liu Y. L. Sun H. Q. (2019). Comparisons of the anti-tumor activity of polysaccharides from fermented mycelia and cultivated fruiting bodies of *Cordyceps militaris* in vitro. Int. J. Biol. Macromol. 130, 307–314. doi: 10.1016/j.ijbiomac.2019.02.155, PMID: 30825564

[ref26] Lorenzo R. Onizuka M. Defrance M. Laurent P. (2020). Combining single-cell RNA-sequencing with a molecular atlas unveils new markers for *Caenorhabditis elegans* neuron classes. Nucleic Acids Res. 48, 7119–7134. doi: 10.1093/nar/gkaa486, PMID: 32542321 PMC7367206

[ref27] Lou H. W. Zhao Y. Chen B. X. Yu Y. H. Tang H. B. Ye Z. W.. (2020). *Cmfhp* gene mediates fruiting body development and carotenoid production in *Cordyceps militaris*. Biomol. Ther. 10, 410–421. doi: 10.3390/biom10030410, PMID: 32155914 PMC7175373

[ref28] Lou H. W. Zhao Y. Tang H. B. Ye Z. W. Wei T. Lin J. F.. (2019). Transcriptome analysis of *Cordyceps militaris* reveals genes associated with carotenoid synthesis and identification of the function of the *cmtns* gene. Front. Microbiol. 10, 2105–2117. doi: 10.3389/fmicb.2019.02105, PMID: 31552008 PMC6746990

[ref29] Luo H. T. He Q. Yang W. He F. Dong J. Hu C. F.. (2023). Single-cell analyses reveal distinct expression patterns and roles of long non-coding RNAs during hESC differentiation into pancreatic progenitors. Stem Cell Res Ther 14, 38–54. doi: 10.1186/s13287-023-03259-x, PMID: 36907881 PMC10010006

[ref30] Mao X. Xing D. Liu D. Xu H. Hou L. Lin P.. (2023). Ecdysteroid UDP-glucosyltransferase expression in *Beauveria bassiana* increases its pathogenicity against early instar silkworm larvae. J. Fungi (Basel) 9, 987–999. doi: 10.3390/jof9100987, PMID: 37888243 PMC10607489

[ref31] Maoka T. (2020). Carotenoids as natural functional pigments. J. Nat. Med. 74, 1–16. doi: 10.1007/s11418-019-01364-x, PMID: 31588965 PMC6949322

[ref32] Matera A. Dulak K. Werner H. Sordon S. Huszcza E. Popłoński J. (2024). Investigation on production and reaction conditions of sucrose synthase based glucosylation cascade towards flavonoid modification. Bioorg. Chem. 146:107287. doi: 10.1016/j.bioorg.2024.107287, PMID: 38503024

[ref33] Miao M. Yu W. Q. Li Y. Sun Y. L. Guo S. D. (2022). Structural elucidation and activities of *Cordyceps militaris*-derived polysaccharides: a review. Front. Nutr. 9, 898674–898693. doi: 10.3389/fnut.2022.898674, PMID: 35711557 PMC9193282

[ref34] Mohideen A. Johansen S. D. Babiak I. (2020). High-throughput identification of adapters in single-read sequencing data. Biomol. Ther. 10, 878–890. doi: 10.3390/biom10060878, PMID: 32521604 PMC7356586

[ref35] Prommaban A. Sriyab S. Marsup P. Neimkhum W. Sirithunyalug J. Anuchapreeda S.. (2022). Comparison of chemical profiles, antioxidation, inhibition of skin extracellular matrix degradation, and anti-tyrosinase activity between mycelium and fruiting body of *Cordyceps militaris* and *Isaria tenuipes*. Pharm. Biol. 60, 225–234. doi: 10.1080/13880209.2021.2025255, PMID: 35068295 PMC8786250

[ref36] Pronozin A. Y. Afonnikov D. A. (2023). ICAnnoLncRNA: a snakemake pipeline for a long non-coding-rna search and annotation in transcriptomic sequences. Genes (Basel) 14, 1131–1151. doi: 10.3390/genes14071331 37510236 PMC10379598

[ref37] Salakhov R. R. Golubenko M. V. Valiakhmetov N. R. Pavlyukova E. N. Zarubin A. A. Babushkina N. P.. (2022). Application of long-read nanopore sequencing to the search for mutations in hypertrophic cardiomyopathy. Int. J. Mol. Sci. 23, 15845–15859. doi: 10.3390/ijms232415845, PMID: 36555486 PMC9779642

[ref38] Shevchuk Y. Kuypers K. Janssens G. E. (2023). Fungi as a source of bioactive molecules for the development of longevity medicines. Ageing Res. Rev. 87, 101929–101942. doi: 10.1016/j.arr.2023.101929, PMID: 37031727

[ref39] Soommat P. Raethong N. Ruengsang R. Thananusak R. Laomettachit T. Laoteng K.. (2024). Light-exposed metabolic responses of *Cordyceps militaris* through transcriptome-integrated genome-scale modeling. Biology (Basel) 13, 139–152. doi: 10.3390/biology13030139, PMID: 38534409 PMC10967962

[ref40] Tan L. Song X. Ren Y. Wang M. Guo C. Guo D.. (2020). Anti-inflammatory effects of cordycepin: a review. Phytother. Res. 2021, 3–17. doi: 10.1002/ptr.6890, PMID: 33090621

[ref41] Tang J. R. Chen G. Lu Y. C. Tang Q. Y. Song W. L. Lin Y.. (2021). Identification of two UDP-glycosyltransferases involved in the main oleanane-type ginsenosides in *Panax japonicus* var. major. Planta 253:91. doi: 10.1007/s00425-021-03617-0, PMID: 33818668

[ref42] Tang H. Chen C. Zou Y. Lou H. Zheng Q. Guo L.. (2019). Purification and structural characterization of a novel natural pigment: cordycepene from edible and medicinal mushroom *Cordyceps militaris*. Appl. Microbiol. Biotechnol. 103, 7943–7952. doi: 10.1007/s00253-019-10101-z, PMID: 31489456

[ref43] Trapnell C. Roberts A. Goff L. Pertea G. Kim D. Kelley D. R.. (2012). Differential gene and transcript expression analysis of RNA-seq experiments with TopHat and cufflinks. Nat. Protoc. 7, 562–578. doi: 10.1038/nprot.2012.016, PMID: 22383036 PMC3334321

[ref44] van Heusden P. Mashologu Z. Lose T. Warren R. Christoffels A. (2022). The COMBAT-TB workbench: making powerful *Mycobacterium tuberculosis* bioinformatics accessible. mSphere 7:e0099121. doi: 10.1128/msphere.00991-21, PMID: 35138128 PMC8827006

[ref45] Vera Alvarez R. Pongor L. S. Mariño-Ramírez L. Landsman D. (2019). TPMCalculator: one-step software to quantify mRNA abundance of genomic features. Bioinformatics 35, 1960–1962. doi: 10.1093/bioinformatics/bty896, PMID: 30379987 PMC6546121

[ref46] Vu T. X. Thai H. D. Dinh B. T. Nguyen H. T. Tran H. T. P. Bui K. T.. (2023). Effects of *MAT1-2* spore ratios on fruiting body formation and degeneration in the heterothallic fungus *Cordyceps militaris*. J. Fungi (Basel) 9, 971–982. doi: 10.3390/jof9100971, PMID: 37888227 PMC10607669

[ref47] Wang X. Peng Z. Wang L. Zhang J. Zhang K. Guo Z.. (2023). *Cordyceps militaris* solid medium extract alleviates lipoteichoic acid-induced MH-S inflammation by inhibiting TLR2/NF-κB/NLRP3 pathways. Int. J. Mol. Sci. 24, 15519–15535. doi: 10.3390/ijms242115519, PMID: 37958501 PMC10648577

[ref48] Wang Y. Yang X. Chen P. Yang S. Zhang H. (2021). Homologous overexpression of genes in *Cordyceps militaris* improves the production of polysaccharides. Food Res. Int. 147, 110452–110460. doi: 10.1016/j.foodres.2021.110452, PMID: 34399454

[ref49] Wongsa B. Raethong N. Chumnanpuen P. Wong-Ekkabut J. Laoteng K. Vongsangnak W. (2020). Alternative metabolic routes in channeling xylose to cordycepin production of *Cordyceps militaris* identified by comparative transcriptome analysis. Genomics 112, 629–636. doi: 10.1016/j.ygeno.2019.04.015, PMID: 31022437

[ref50] Wu B. Cao X. Liu H. Zhu C. Klee H. Zhang B.. (2019). UDP-glucosyltransferase PpUGT85A2 controls volatile glycosylation in peach. J. Exp. Bot. 70, 925–936. doi: 10.1093/jxb/ery419, PMID: 30481327 PMC6363097

[ref51] Wu N. Ge X. Yin X. Yang L. Chen L. Shao R.. (2024). A review on polysaccharide biosynthesis in *Cordyceps militaris*. Int. J. Biol. Macromol. 260, 129336–129350. doi: 10.1016/j.ijbiomac.2024.129336, PMID: 38224811

[ref52] Xia Y. Luo F. Shang Y. Chen P. Lu Y. Wang C. (2017). Fungal cordycepin biosynthesis is coupled with the production of the safeguard molecule pentostatin. Cell Chem. Biol. 24, 1479–1489.e4. doi: 10.1016/j.chembiol.2017.09.001, PMID: 29056419

[ref53] Xiang L. Li Y. Zhu Y. Luo H. Li C. Xu X.. (2014). Transcriptome analysis of the *Ophiocordyceps sinensis* fruiting body reveals putative genes involved in fruiting body development and cordycepin biosynthesis. Genomics 103, 154–159. doi: 10.1016/j.ygeno.2014.01.002, PMID: 24440419

[ref54] Xu D. Yan B. Yang S. Zhang H. (2023). Overexpression of glycosyltransferase genes enhance the production of extracellular polysaccharides in Cordyceps militaris. Research Square. May 24 (Version 1), 1–18. doi: 10.21203/rs.3.rs-2961548/v1

[ref55] Xu Z. Pu X. Gao R. Demurtas O. C. Fleck S. J. Richter M.. (2020). Tandem gene duplications drive divergent evolution of caffeine and crocin biosynthetic pathways in plants. BMC Biol. 18, 63–73. doi: 10.1186/s12915-020-00795-3, PMID: 32552824 PMC7302004

[ref56] Yang T. Guo M. Yang H. Guo S. Dong C. (2016). The blue-light receptor *CmWC-1* mediates fruit body development and secondary metabolism in *Cordyceps militaris*. Appl. Microbiol. Biotechnol. 100, 743–755. doi: 10.1007/s00253-015-7047-6, PMID: 26476643

[ref57] Yuan X. Li R. He W. Xu W. Xu W. Yan G.. (2024). Progress in identification of UDP-glycosyltransferases for ginsenoside biosynthesis. J. Nat. Prod. 87, 1246–1267. doi: 10.1021/acs.jnatprod.3c00630, PMID: 38449105

[ref59] Zhang H. Wang Y. X. Tong X. X. Yokoyama W. Cao J. Wang F.. (2020). Overexpression of ribonucleotide reductase small subunit, *RNRM*, increases cordycepin biosynthesis in transformed *Cordyceps militaris*. Chin. J. Nat. Med. 18, 393–400. doi: 10.1016/S1875-5364(20)30046-7, PMID: 32451097

[ref60] Zhang M. Q. Yang Z. Dong Y. X. Zhu Y. L. Chen X. Y. Dai C. C.. (2024). Expression of endogenous UDP-glucosyltransferase in endophyte phomopsis liquidambaris reduces deoxynivalenol contamination in wheat. Fungal Genet. Biol. 173:103899. doi: 10.1016/j.fgb.2024.103899, PMID: 38802054

[ref61] Zhao Y. Li S. L. Chen H. Y. Zou Y. Zheng Q. W. Guo L. Q.. (2021). Enhancement of carotenoid production and its regulation in edible mushroom *Cordyceps militaris* by abiotic stresses. Enzym. Microb. Technol. 148:109808. doi: 10.1016/j.enzmictec.2021.109808, PMID: 34116757

[ref63] Zheng P. Xia Y. Xiao G. Xiong C. Hu X. Zhang S.. (2011). Genome sequence of the insect pathogenic fungus *Cordyceps militaris*, a valued traditional Chinese medicine. Genome Biol. 12:R116. doi: 10.1186/gb-2011-12-11-r116, PMID: 22112802 PMC3334602

