# Supplementary material for: Discovering a novel glycosyltransferase gene CmUGT1 enhances main metabolites production of Cordyceps militaris
Source: Front Microbiol. 2024 Oct 22;15:1437963. doi: 10.3389/fmicb.2024.1437963 (PMC11534717; doi:10.3389/fmicb.2024.1437963)
Supplement: Supplementary file 1 [file Data_Sheet_1.docx]

Supplementary Materials

Discovering a novel glycosyltransferase gene *CmUGT1* enhances main metabolites production of *Cordyceps militaris*

Rong-an He^1 a^, Chen Huang^2 a^, Chun-hui Zheng^1 a^, Jing Wang^1^, Si-wen Yuan^1^, Bai-xiong Chen^1^*, Kun Feng^1^*

Correspondence: Bai-xiong Chen, Kun Feng

E-mail: baixiong@zmu.edu.cn(BXC); fengk@zmu.edu.cn(KF)

# Figure S1 The PCR duplication of four different developmental stages of *C. militaris.*

# Figure S2 The uniform distribution of four different developmental stages of *C. militaris.*

**Figure S3** GO analysis of CM2/CM1.

**Figure S4** GO analysis of CM3/CM2.

**Figure S5** GO analysis of CM4/CM3.

**Figure S6** KEGG analysis of CM2/CM1.

**Figure S7** KEGG analysis of CM3/CM2.

**Figure S8** KEGG analysis of CM4/CM3.

# Figure S9 Colony PCR and enzyme digestion identification of recombinant plasmid.

# Figure S10 PCR identification of overexpressed transformants from *C. militaris*.

**Figure S11** The UPLC chromatographic peak of cordycepin in supernatant of *C. militaris.*

**Figure S12** The UPLC chromatographic peak of cordycepin in mycelium of *C. militaris.*

**Table S1** Sequencing raw data of four different developmental stages of *C. militaris.*

**Table S2** Clean data of four different developmental stages of *C. militaris.*

**Table S3** Analysis of GO enrichment of CM2/CM1.

**Table S4** Analysis of GO enrichment of CM3/CM2.

**Table S5** Analysis of GO enrichment of CM4/CM3.

**Table S6** Analysis of KEGG enrichment of CM2/CM1.

**Table S7** Analysis of KEGG enrichment of CM3/CM2.

**Table S8** Analysis of KEGG enrichment of CM4/CM3.

**Table S9** Carotenoid content in mycelium of *C. militaris*.

**Table S10** Polysaccharides content in supernatant of *C. militaris*.

**Table S11** Cordycepin content in supernatant of *C. militaris*.

**Table S12** Cordycepin content in mycelium of *C. militaris*.


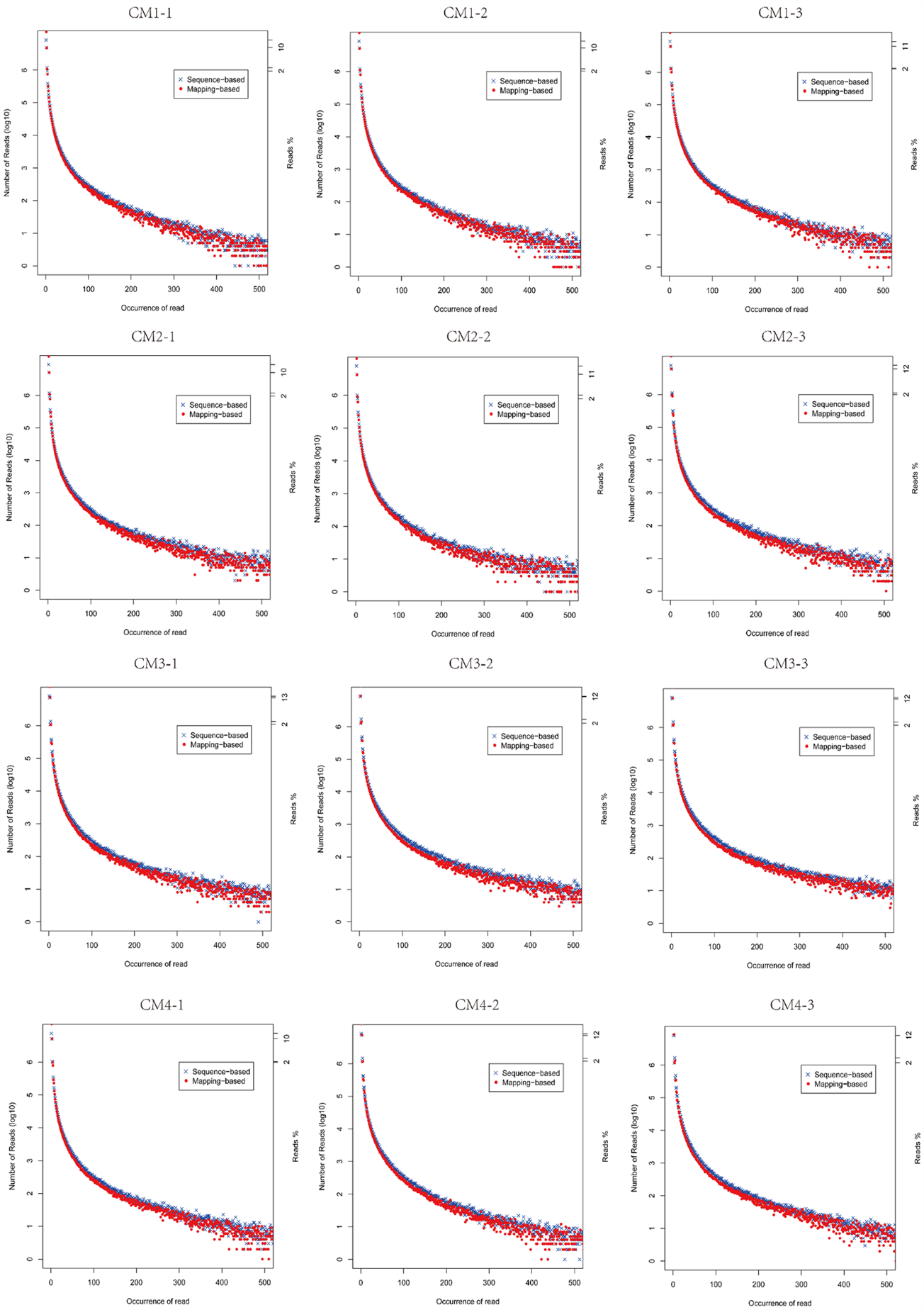


**Figure S1** The PCR duplication of four different developmental stages of *C. militaris*


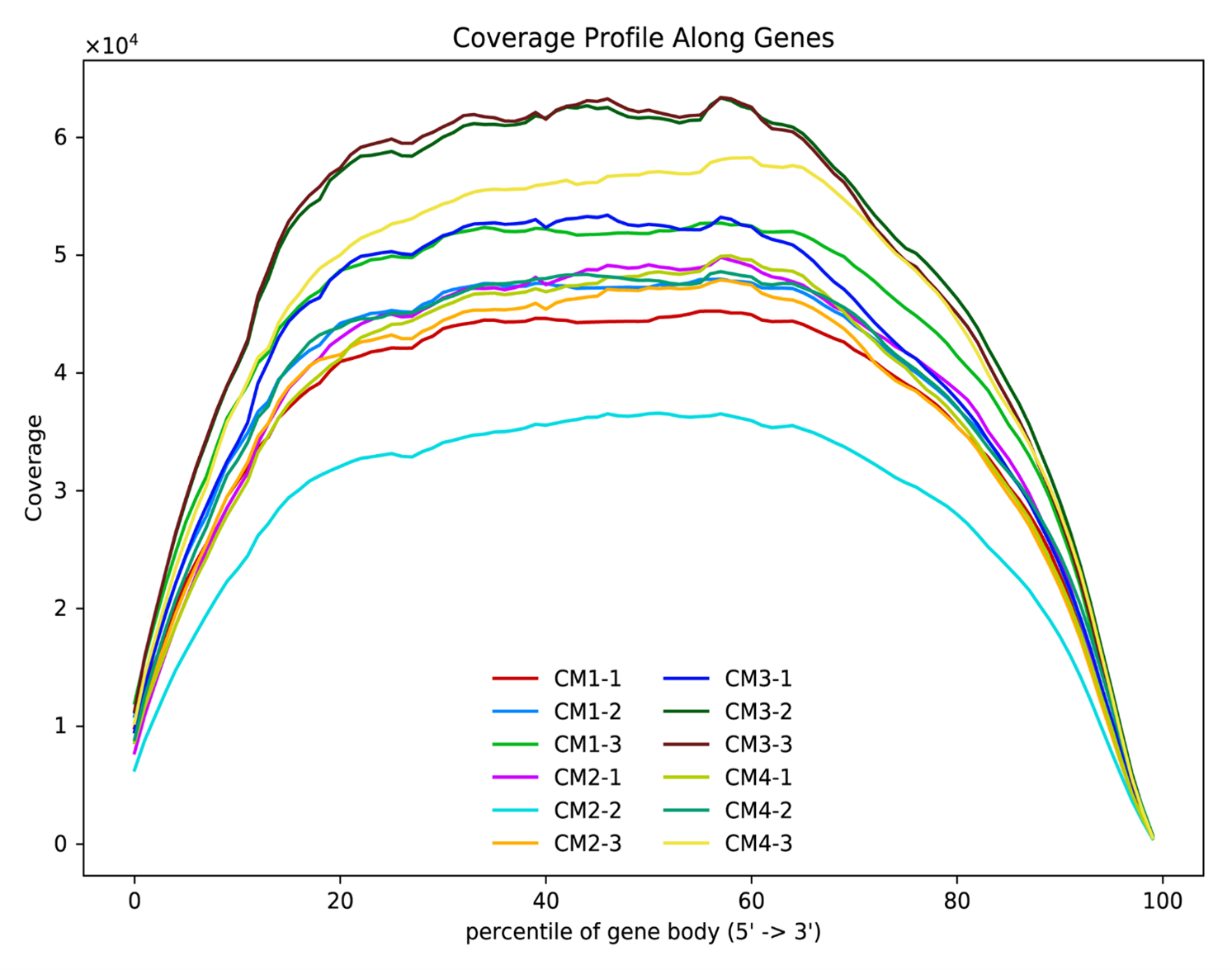


**Figure S2** The uniform distribution of four different developmental stages of *C. militaris*


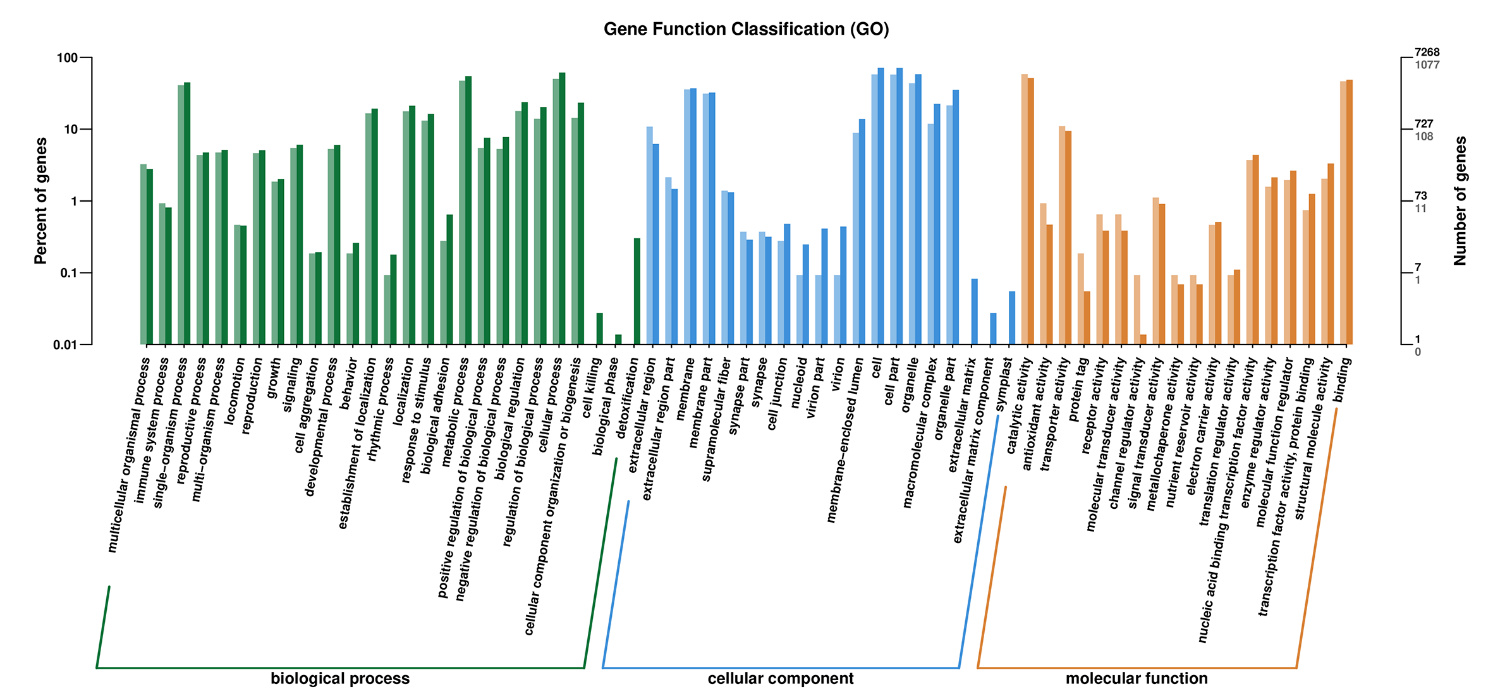


**Figure S3** GO analysis of CM2/CM1.


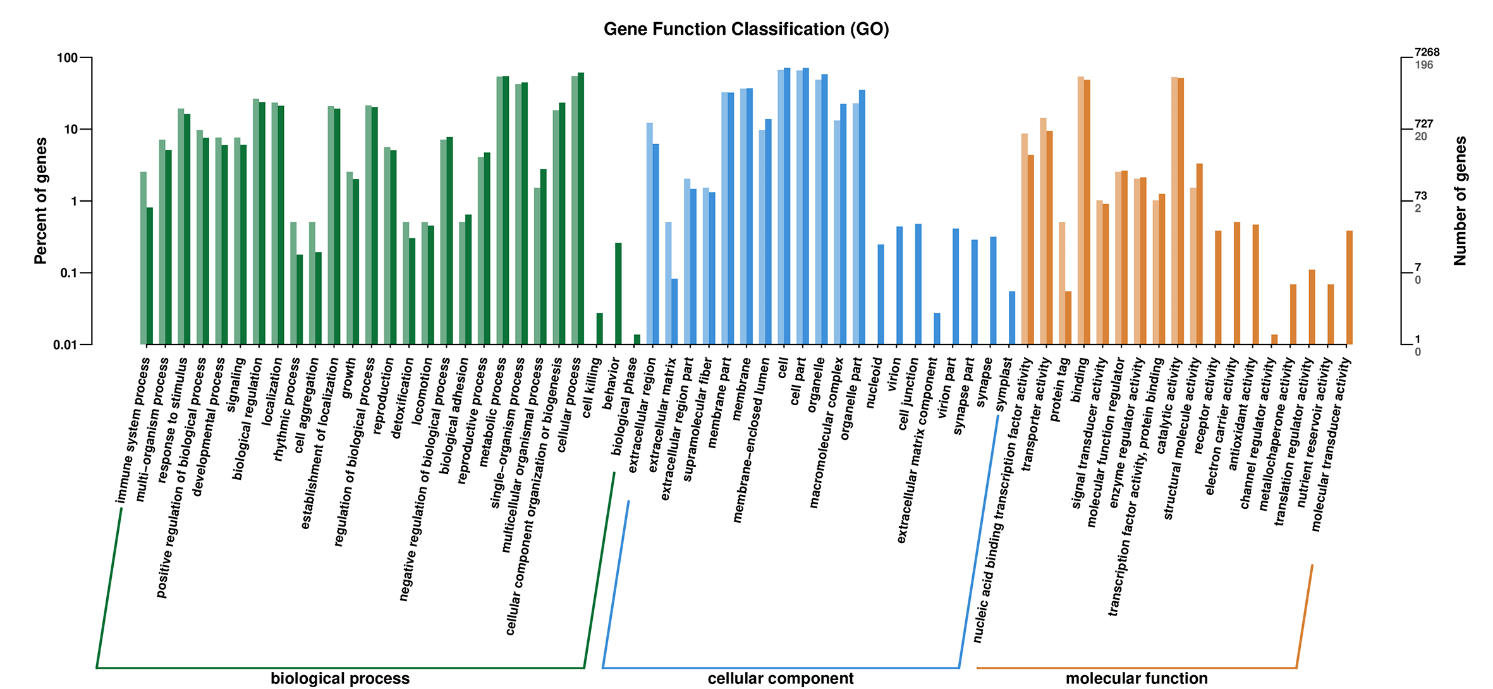


**Figure S4** GO analysis of CM3/CM2.

*
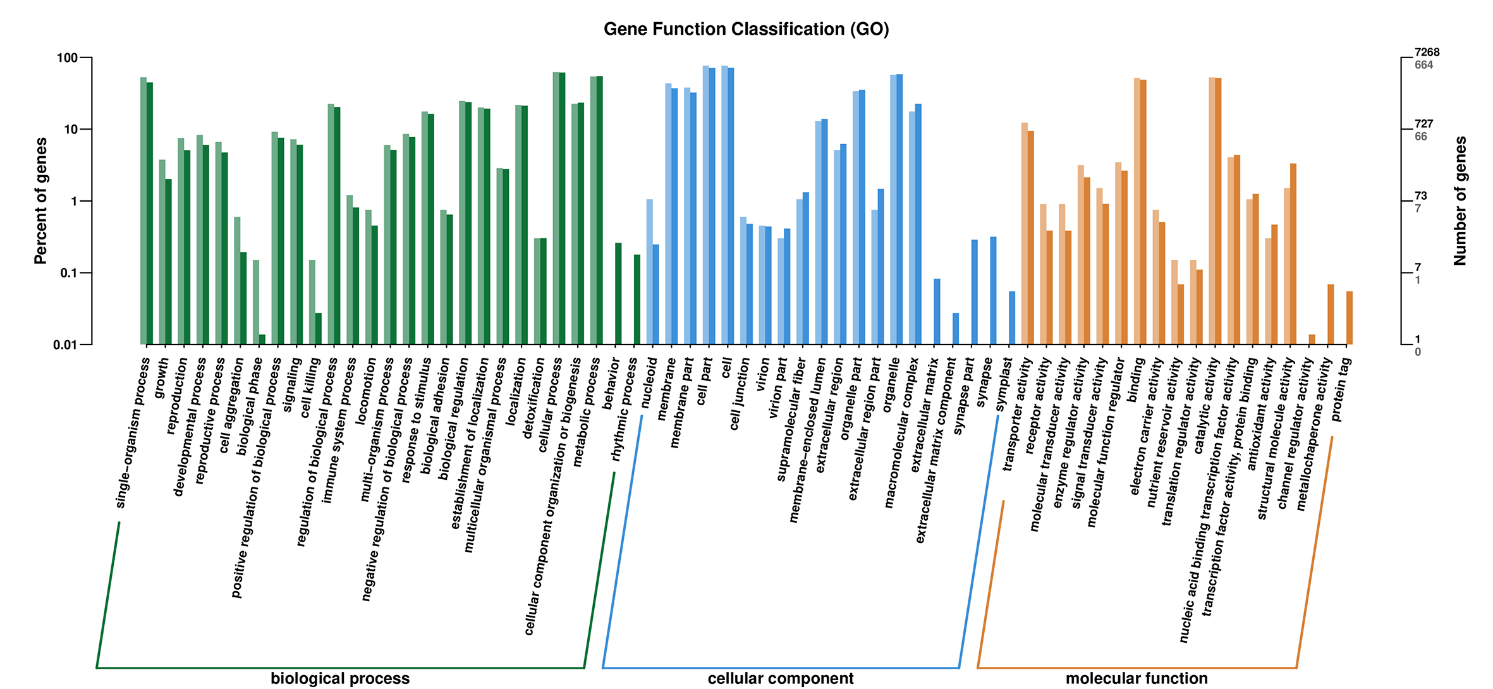
*

**Figure S5** GO analysis of CM4/CM3.

**Figure S6** KEGG analysis of CM2/CM1.

**Figure S7** KEGG analysis of CM3/CM2.

**Figure S8** KEGG analysis of CM4/CM3.

**Figure S9** Colony PCR and enzyme digestion identification of recombinant plasmid. (A) The identification of p390-BlpR-Pcmef1-CmUGT1 colony PCR (M: marker 100~2000 bp, 1~2: the identification of *CmUGT1* using primer 1083+1310: 1719 bp). (B) The identification of enzyme digestion (M: marker 100~5000 bp. 1,3: recombinant plasmid not digested; 2,4: recombinant plasmid digested by *Eco*RI and *Not*I in 37℃, 30 min, 5692 bp, 3088 bp, 1532 bp, 1290 bp).


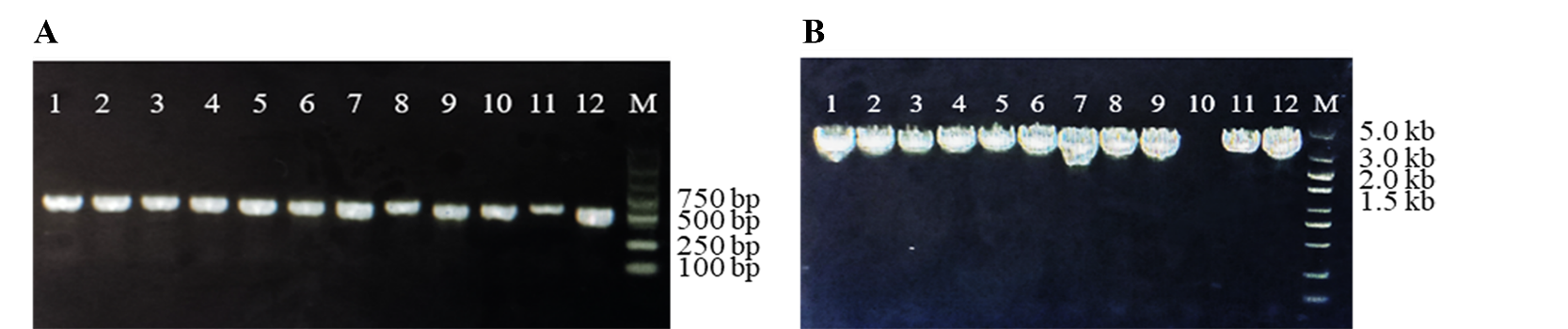


**Figure S10** PCR identification of overexpressed transformants from *C. militaris*. (A) The identification of preliminary screening (M: marker 100~5000 bp, 1~12: the identification of BlpR). (B) The identification of complete expression box (M: marker 100~5000 bp, 1~12: the identification of complete expression box).

**Figure S11** The UPLC chromatographic peak of cordycepin in supernatant of *C. militaris.* (A) Cordycepin standard. (B)Wild type. (C) *CmUGT1*-overexpressing type. The mobile phase consists of 85% ultrapure water and 15% (v/v) methanol, flow rate of 2 mL/min, column temperature of 40℃, detection wavelength of 260 nm. The sample was injected with 2 μL each time.

**Figure S12** The UPLC chromatographic peak of cordycepin in mycelium of *C. militaris*. (A) Cordycepin standard. (B)Wild type. (C) *CmUGT1*-overexpressing type. The mobile phase consists of 85% ultrapure water and 15% (v/v) methanol, flow rate of 2 mL/min, column temperature of 40℃, detection wavelength of 260 nm. The sample was injected with 2 μL each time.

**Table S1** Sequencing raw data of four different developmental stages of *C. militaris*

|  | **CM1-1** | **CM1-2** | **CM1-3** | **CM2-1** | **CM2-2** | **CM2-3** | **CM3-1** | **CM3-2** | **CM3-3** | **CM4-1** | **CM4-2** | **CM4-3** |
| --- | --- | --- | --- | --- | --- | --- | --- | --- | --- | --- | --- | --- |
| Total Reads Count(#) | 50986174 | 52894006 | 61301252 | 55160354 | 49027456 | 62234430 | 60993494 | 84882096 | 68782968 | 53016092 | 62371292 | 68644780 |
| Total Bases Count(bp) | 7345003899 | 7604523139 | 8741237013 | 7900186613 | 7032918923 | 8631927355 | 8431045374 | 11636416453 | 9504948752 | 7528726498 | 8658216792 | 9406728244 |
| Average Read Length(bp) | 144.06 | 143.77 | 142.59 | 143.22 | 143.45 | 138.70 | 138.23 | 137.09 | 138.19 | 142.01 | 138.82 | 137.03 |
| Q10 Bases Count(bp) | 7344981822 | 7604499738 | 8741209924 | 7900162370 | 7032897328 | 8631900261 | 8431018815 | 11636379548 | 9504918620 | 7528703251 | 8658189471 | 9406698412 |
| Q10 Bases Ratio(%) | 100.00% | 100.00% | 100.00% | 100.00% | 100.00% | 100.00% | 100.00% | 100.00% | 100.00% | 100.00% | 100.00% | 100.00% |
| Q20 Bases Count(bp) | 7278859004 | 7536095822 | 8666866410 | 7814673170 | 6964922331 | 8549219098 | 8361644521 | 11540688662 | 9424670357 | 7452525397 | 8582368654 | 9327367957 |
| Q20 Bases Ratio(%) | 99.10% | 99.10% | 99.15% | 98.92% | 99.03% | 99.04% | 99.18% | 99.18% | 99.16% | 98.99% | 99.12% | 99.16% |
| Q30 Bases Count(bp) | 7085479604 | 7336195047 | 8447373364 | 7576952656 | 6767687074 | 8310901462 | 8156073096 | 11254431143 | 9188694154 | 7237092188 | 8358821269 | 9091467885 |
| Q30 Bases Ratio(%) | 96.47% | 96.47% | 96.64% | 95.91% | 96.23% | 96.28% | 96.74% | 96.72% | 96.67% | 96.13% | 96.54% | 96.65% |
| N Bases Count(bp) | 22077 | 23401 | 27089 | 24243 | 21595 | 27094 | 26559 | 36905 | 30132 | 23247 | 27321 | 29832 |
| N Bases Ratio(%) | 0.00% | 0.00% | 0.00% | 0.00% | 0.00% | 0.00% | 0.00% | 0.00% | 0.00% | 0.00% | 0.00% | 0.00% |
| GC Bases Count(bp) | 4256465526 | 4415106784 | 5062997803 | 4510429817 | 3846523786 | 4846844715 | 4798508599 | 6530772433 | 5443754974 | 4327492743 | 4923806246 | 5380036827 |
| GC Bases Ratio(%) | 57.95% | 58.06% | 57.92% | 57.09% | 54.69% | 56.15% | 56.91% | 56.12% | 57.27% | 57.48% | 56.87% | 57.19% |

**Table S2** Clean data of four different developmental stages of *C. militaris.*

|  | **CM1-1** | **CM1-2** | **CM1-3** | **CM2-1** | **CM2-2** | **CM2-3** | **CM3-1** | **CM3-2** | **CM3-3** | **CM4-1** | **CM4-2** | **CM4-3** |
| --- | --- | --- | --- | --- | --- | --- | --- | --- | --- | --- | --- | --- |
| Total reads | 49723476 | 52023540 | 60399170 | 53845192 | 46645036 | 52917054 | 58928450 | 73560890 | 67972272 | 51245964 | 61593134 | 67929252 |
| Total mapped | 48065693 | 50661120 | 58894528 | 51768820 | 39861299 | 50282867 | 56734270 | 70072900 | 65729073 | 49637423 | 60112692 | 66373116 |
| Mutiple mapped | 111677 | 122695 | 137677 | 130841 | 79502 | 130241 | 181237 | 237361 | 191750 | 125403 | 119485 | 119514 |
| Uniquely mapped | 47954016 | 50538425 | 58756851 | 51637979 | 39781797 | 50152626 | 56553033 | 69835539 | 65537323 | 49512020 | 59993207 | 66253602 |
| Read-1 mapped | 24018843 | 25305343 | 29421233 | 25870839 | 19929712 | 25126401 | 28313699 | 34961833 | 32823400 | 24794736 | 30034760 | 33179142 |
| Read-2 mapped | 23935173 | 25233082 | 29335618 | 25767140 | 19852085 | 25026225 | 28239334 | 34873706 | 32713923 | 24717284 | 29958447 | 33074460 |
| Reads map to '+' | 23959300 | 25251700 | 29361207 | 25779100 | 19868429 | 25057868 | 28243852 | 34885371 | 32741256 | 24724725 | 29981972 | 33117175 |
| Reads map to '-' | 23994716 | 25286725 | 29395644 | 25858879 | 19913368 | 25094758 | 28309181 | 34950168 | 32796067 | 24787295 | 30011235 | 33136427 |
| Non-splice reads | 40694986 | 42520563 | 50008370 | 44618218 | 34217594 | 42584589 | 47639543 | 59395604 | 55545723 | 41994441 | 52317607 | 57274909 |
| Splice reads | 7259030 | 8017862 | 8748481 | 7019761 | 5564203 | 7568037 | 8913490 | 10439935 | 9991600 | 7517579 | 7675600 | 8978693 |
| Reads mapped in proper pairs | 46312290 | 48526058 | 56453844 | 49529494 | 38242448 | 47202544 | 53289662 | 65588140 | 61837054 | 47327006 | 56698788 | 62576402 |

**Table S3** Analysis of GO enrichment of CM2/CM1

| **GO.ID** | **Term** | **Ontology** | **Significant** | **Annotated** | **Pvalue** | **log_2_FoldChange** |
| --- | --- | --- | --- | --- | --- | --- |
| GO:0019748 | secondary metabolic process | biological process | 22/733 | 82/5524 | 0.00074 | 4.17 |
| GO:0055085 | transmembrane transport | biological process | 98/733 | 548/5524 | 0.00074 | 3.96 |
| GO:0010499 | proteasomal ubiquitin-independent protein catabolic process | biological process | 9/733 | 15/5524 | 2.90E-05 | 1.87 |
| GO:0042026 | protein refolding | biological process | 6/733 | 9/5524 | 0.00031 | 1.79 |
| GO:0046323 | glucose import | biological process | 11/733 | 27/5524 | 0.00036 | 1.63 |
| GO:0050773 | regulation of dendrite development | biological process | 5/733 | 7/5524 | 0.00068 | 1.32 |
| GO:0015758 | glucose transport | biological process | 11/733 | 27/5524 | 0.00036 | / |
| GO:0008645 | hexose transport | biological process | 11/733 | 29/5524 | 0.00074 | / |
| GO:0005576 | extracellular region | cellular component | 117/843 | 453/6239 | 2.30E-13 | 4.05 |
| GO:0071944 | cell periphery | cellular component | 136/843 | 811/6239 | 0.00263 | 2.15 |
| GO:0005886 | plasma membrane | cellular component | 108/843 | 630/6239 | 0.00367 | 2.12 |
| GO:0005618 | cell wall | cellular component | 21/843 | 84/6239 | 0.00319 | 2.07 |
| GO:0031225 | anchored component of membrane | cellular component | 15/843 | 57/6239 | 0.00719 | 1.90 |
| GO:0019773 | proteasome core complex, alpha-subunit complex | cellular component | 5/843 | 7/6239 | 0.00074 | 1.63 |
| GO:0005839 | proteasome core complex | cellular component | 9/843 | 15/6239 | 3.40E-05 | 1.63 |
| GO:0030312 | external encapsulating structure | cellular component | 21/843 | 84/6239 | 0.00319 | / |
| GO:0016705 | oxidoreductase activity, | molecular function | 41/902 | 146/5857 | 5.40E-05 | 6.54 |
| GO:0004497 | monooxygenase activity | molecular function | 40/902 | 139/5857 | 3.60E-05 | 5.98 |
| GO:0003824 | catalytic activity | molecular function | 630/902 | 3766/5857 | 7.90E-05 | 5.60 |
| GO:0050661 | NADP binding | molecular function | 19/902 | 59/5857 | 0.00092 | 4.82 |
| GO:0016491 | oxidoreductase activity | molecular function | 181/902 | 811/5857 | 1.10E-08 | 1.90 |
| GO:0004298 | threonine-type endopeptidase activity | molecular function | 10/902 | 15/5857 | 1.00E-05 | 1.87 |
| GO:0004175 | endopeptidase activity | molecular function | 43/902 | 149/5857 | 1.70E-05 | 1.07 |
| GO:0070003 | threonine-type peptidase activity | molecular function | 10/902 | 15/5857 | 1.00E-05 | / |

**Table S4** Analysis of GO enrichment of CM3/CM2

| **GO.ID** | **Term** | **Ontology** | **Significant** | **Annotated** | **Pvalue** | **log_2_FoldChange** |
| --- | --- | --- | --- | --- | --- | --- |
| GO:0005975 | carbohydrate metabolic process | biological process | 24/152 | 367/5524 | 5.30E-05 | 2.87 |
| GO:0006817 | phosphate ion transport | biological process | 4/152 | 13/5524 | 0.00032 | 2.63 |
| GO:0045087 | innate immune response | biological process | 5/152 | 25/5524 | 0.0005 | 1.72 |
| GO:0016052 | carbohydrate catabolic process | biological process | 14/152 | 165/5524 | 0.00016 | 1.18 |
| GO:0000272 | polysaccharide catabolic process | biological process | 10/152 | 91/5524 | 0.00018 | 1.01 |
| GO:0015698 | inorganic anion transport | biological process | 5/152 | 22/5524 | 0.00027 | / |
| GO:0005976 | polysaccharide metabolic process | biological process | 12/152 | 140/5524 | 0.00043 | / |
| GO:0070838 | divalent metal ion transport | biological process | 7/152 | 64/5524 | 0.00173 | / |
| GO:0005887 | integral component of plasma membrane | cellular component | 14/168 | 183/6239 | 0.00038 | 1.96 |
| GO:0005576 | extracellular region | cellular component | 24/168 | 453/6239 | 0.00098 | 1.9 |
| GO:0019005 | SCF ubiquitin ligase complex | cellular component | 3/168 | 13/6239 | 0.0045 | 1.42 |
| GO:0097038 | perinuclear endoplasmic reticulum | cellular component | 2/168 | 7/6239 | 0.01385 | 1.29 |
| GO:0000131 | incipient cellular bud site | cellular component | 3/168 | 18/6239 | 0.01162 | 1.26 |
| GO:0000407 | pre-autophagosomal structure | cellular component | 3/168 | 19/6239 | 0.01353 | 1.09 |
| GO:0031226 | intrinsic component of plasma membrane | cellular component | 15/168 | 197/6239 | 0.00025 | / |
| GO:0044459 | plasma membrane part | cellular component | 15/168 | 252/6239 | 0.0031 | / |
| GO:0005315 | inorganic phosphate transmembrane transporter activity | molecular function | 4/170 | 10/5857 | 0.00013 | 2.63 |
| GO:0046873 | metal ion transmembrane transporter activity | molecular function | 11/170 | 92/5857 | 6.20E-05 | 1.81 |
| GO:0016798 | hydrolase activity, acting on glycosyl bonds | molecular function | 18/170 | 180/5857 | 3.80E-06 | 1.59 |
| GO:0030246 | carbohydrate binding | molecular function | 12/170 | 84/5857 | 4.50E-06 | 1.58 |
| GO:0004553 | hydrolase activity, hydrolyzing O-glycosyl compounds | molecular function | 16/170 | 152/5857 | 6.90E-06 | 1.58 |
| GO:0004620 | phospholipase activity | molecular function | 6/170 | 27/5857 | 9.80E-05 | 1 |
| GO:0004629 | phospholipase C activity | molecular function | 4/170 | 8/5857 | 4.40E-05 | / |
| GO:0001871 | pattern binding | molecular function | 4/170 | 11/5857 | 0.00019 | / |

**Table S5** Analysis of GO enrichment of CM4/CM3

| **GO.ID** | | **Term** | | **Ontology** | | | **Significant** | | **Annotated** | | | **Pvalue** | **log_2_FoldChange** |
| --- | --- | --- | --- | --- | --- | --- | --- | --- | --- | --- | --- | --- | --- |
| GO:0008610 | | lipid biosynthetic process | | biological process | | | 51/521 | | 255/5524 | | | 1.00E-07 | 5.34 |
| GO:0044711 | | single-organism biosynthetic process | | biological process | | | 110/521 | | 751/5524 | | | 4.40E-07 | 2.34 |
| GO:0046165 | | alcohol biosynthetic process | | biological process | | | 19/521 | | 59/5524 | | | 9.10E-07 | 2.25 |
| GO:0044283 | | small molecule biosynthetic process | | biological process | | | 62/521 | | 366/5524 | | | 2.10E-06 | 1.97 |
| GO:0006696 | | ergosterol biosynthetic process | | biological process | | | 12/521 | | 30/5524 | | | 7.70E-06 | 1.71 |
| GO:0044108 | | cellular alcohol biosynthetic process | | biological process | | | 12/521 | | 30/5524 | | | 7.70E-06 | 1.26 |
| GO:1901617 | | organic hydroxy compound biosynthetic process | | biological process | | | 25/521 | | 102/5524 | | | 5.40E-06 | / |
| GO:1990204 | | oxidoreductase complex | | cellular component | | | 14/597 | | 39/6239 | | | 7.30E-06 | 3.22 |
| GO:0051286 | | cell tip | | cellular component | | | 24/597 | | 132/6239 | | | 0.00144 | 1.28 |
| GO:0000786 | | nucleosome | | cellular component | | | 4/597 | | 6/6239 | | | 0.00106 | 0.91 |
| GO:0032993 | | protein-DNA complex | | cellular component | | | 12/597 | | 48/6239 | | | 0.00146 | -0.61 |
| GO:0016021 | | integral component of membrane | | cellular component | | | 228/597 | | 2061/6239 | | | 0.00301 | -1.27 |
| GO:0009295 | | nucleoid | | cellular component | | | 7/597 | | 18/6239 | | | 0.00088 | / |
| GO:0005886 | | plasma membrane | | cellular component | | | 83/597 | | 630/6239 | | | 0.00113 | / |
| GO:0031224 | | intrinsic component of membrane | | cellular component | | | 233/597 | | 2109/6239 | | | 0.00282 | / |
| GO:0016758 | | transferase activity, transferring hexosyl groups | | molecular function | | | 19/546 | | 88/5857 | | | 0.00037 | 2.42 |
| GO:0004584 | | dolichyl-phosphate-mannose-glycolipid alpha-mannosyltransferase activity | | molecular function | | | 3/546 | | 3/5857 | | | 0.00081 | 2.08 |
| GO:0030170 | | pyridoxal phosphate binding | | molecular function | | | 15/546 | | 69/5857 | | | 0.0014 | 1.9 |
| GO:0016597 | | amino acid binding | | molecular function | | | 6/546 | | 16/5857 | | | 0.00226 | 0.98 |
| GO:0046912 | | transferase activity | | molecular function | | | 5/546 | | 9/5857 | | | 0.00063 | 0.29 |
| GO:0008484 | | sulfuric ester hydrolase activity | | molecular function | | | 5/546 | | 10/5857 | | | 0.00117 | -0.56 |
| GO:0070279 | | vitamin B6 binding | | molecular function | | | 15/546 | | 69/5857 | | | 0.0014 | / |
| GO:0004774 | | succinate-CoA ligase activity | | molecular function | | | 3/546 | | 4/5857 | | | 0.003 | / |
|  | |  | |  | |  |  | |  | |  |  |  |
|  | |  | |  | |  |  | |  | |  |  |  |
|  | |  | |  | |  |  | |  | |  |  |  |
|  | |  | |  | |  |  | |  | |  |  |  |
|  | |  | |  | |  |  | |  | |  |  |  |
|  | |  | |  | |  |  | |  | |  |  |  |
|  | |  | |  | |  |  | |  | |  |  |  |

**Table S6** Analysis of KEGG enrichment of CM2/CM1

| **ID** | | **Description** | | **Significant** | **Annotated** | | **Pvalue** | | | **log_2_FoldChange** | |
| --- | --- | --- | --- | --- | --- | --- | --- | --- | --- | --- | --- |
| ko00052 | | Galactose metabolism | | 7/243 | 23/2046 | | 0.013997758 | | | 5.94 | |
| ko00460 | | Cyanoamino acid metabolism | | 7/243 | 20/2046 | | 0.006050669 | | | 5.55 | |
| ko00071 | | Fatty acid degradation | | 9/243 | 23/2046 | | 0.000736841 | | | 5.31 | |
| ko00380 | | Tryptophan metabolism | | 12/243 | 38/2046 | | 0.000951448 | | | 5.31 | |
| ko01200 | | Carbon metabolism | | 23/243 | 108/2046 | | 0.002931466 | | | 4.51 | |
| ko00680 | | Methane metabolism | | 7/243 | 22/2046 | | 0.010804729 | | | 4.51 | |
| ko00980 | | Metabolism of xenobiotics by cytochrome P450 | | 7/243 | 15/2046 | | 0.000849235 | | | 4.29 | |
| ko00982 | | Drug metabolism - cytochrome P450 | | 7/243 | 15/2046 | | 0.000849235 | | | 4.29 | |
| ko01220 | | Degradation of aromatic compounds | | 8/243 | 20/2046 | | 0.001239549 | | | 4.29 | |
| ko00010 | | Glycolysis / Gluconeogenesis | | 12/243 | 41/2046 | | 0.001999021 | | | 4.29 | |
| ko00830 | | Retinol metabolism | | 3/243 | 5/2046 | | 0.013788027 | | | 4.29 | |
| ko00930 | | Caprolactam degradation | | 5/243 | 8/2046 | | 0.000939767 | | | 4.17 | |
| ko00500 | | Starch and sucrose metabolism | | 10/243 | 37/2046 | | 0.008731491 | | | 4.02 | |
| ko00051 | | Fructose and mannose metabolism | | 11/243 | 32/2046 | | 0.000695993 | | | 3.95 | |
| ko00940 | | Phenylpropanoid biosynthesis | | 6/243 | 10/2046 | | 0.000367223 | | | 3.37 | |
| ko00410 | | beta-Alanine metabolism | | 8/243 | 23/2046 | | 0.003506377 | | | 2.17 | |
| ko03050 | | Proteasome | | 12/243 | 34/2046 | | 0.000298803 | | | 1.87 | |
| ko00232 | | Caffeine metabolism | | 2/243 | 2/2046 | | 0.014054736 | | | 1.84 | |
| ko00350 | | Tyrosine metabolism | | 11/243 | 30/2046 | | 0.000366607 | | | / | |
| ko00965 | | Betalain biosynthesis | | 4/243 | 5/2046 | | 0.000882217 | | | / | |
| ko00950 | | Isoquinoline alkaloid biosynthesis | | 6/243 | 12/2046 | | 0.001312584 | | | / | |
|  | |  | |  | | |  | |  |  | |
|  | |  | |  | | |  | |  |  | |
|  | |  | |  | | |  | |  |  | |
|  | |  | |  | | |  | |  |  | |
|  | |  | |  | | |  | |  |  | |
|  | |  | |  | | |  | |  |  | |
|  | |  | |  | | |  | |  |  | |
|  | |  | |  | | |  | |  |  | |
|  | |  | |  | | |  | |  |  | |
|  | |  | |  | | |  | |  |  | |
|  | |  | |  | | |  | |  |  | |
|  | |  | |  | | |  | |  |  | |
|  | |  | |  | | |  | |  |  | |
|  | |  | |  | | |  | |  |  | |
|  | |  | |  | | |  | |  |  | |
|  | |  | |  | | |  | |  |  | |
|  | |  | |  | | |  | |  |  | |
|  | |  | |  | | |  | |  |  | |
|  | |  | |  | | |  | |  |  | |
|  | |  | |  | | |  | |  |  | |
|  | |  | |  | | |  | |  |  | |

**Table S7** Analysis of KEGG enrichment of CM3/CM2

| **ID** | | **Description** | | **Significant** | **Annotated** | | | **Pvalue** | | **log_2_FoldChange** | |
| --- | --- | --- | --- | --- | --- | --- | --- | --- | --- | --- | --- |
| ko00520 | | Amino sugar and nucleotide sugar metabolism | | 3/47 | 54/2046 | | | 0.125059 | | 2.87 | |
| ko00564 | | Glycerophospholipid metabolism | | 5/47 | 47/2046 | | | 0.003834 | | 2.03 | |
| ko00565 | | Ether lipid metabolism | | 3/47 | 15/2046 | | | 0.004261 | | 2.03 | |
| ko02024 | | Quorum sensing | | 3/47 | 17/2046 | | | 0.006165 | | 2.03 | |
| ko04390 | | Hippo signaling pathway | | 2/47 | 14/2046 | | | 0.039436 | | 2.03 | |
| ko00562 | | Inositol phosphate metabolism | | 2/47 | 25/2046 | | | 0.110977 | | 2.03 | |
| ko04392 | | Hippo signaling pathway - multiple species | | 1/47 | 6/2046 | | | 0.130303 | | 2.01 | |
| ko00500 | | Starch and sucrose metabolism | | 5/47 | 37/2046 | | | 0.001294 | | 1.83 | |
| ko00052 | | Galactose metabolism | | 5/47 | 23/2046 | | | 0.000127 | | 1.68 | |
| ko00511 | | Other glycan degradation | | 3/47 | 11/2046 | | | 0.001648 | | 1.68 | |
| ko00600 | | Sphingolipid metabolism | | 3/47 | 26/2046 | | | 0.020401 | | 1.68 | |
| ko00450 | | Selenocompound metabolism | | 2/47 | 9/2046 | | | 0.016781 | | 1.38 | |
| ko00592 | | alpha-Linolenic acid metabolism | | 1/47 | 4/2046 | | | 0.088832 | | 1.33 | |
| ko00590 | | Arachidonic acid metabolism | | 1/47 | 5/2046 | | | 0.109804 | | 1.33 | |
| ko00591 | | Linoleic acid metabolism | | 1/47 | 2/2046 | | | 0.045427 | | 1.32 | |
| ko03060 | | Protein export | | 2/47 | 17/2046 | | | 0.056431 | | 1.21 | |
| ko04141 | | Protein processing in endoplasmic reticulum | | 4/47 | 74/2046 | | | 0.087255 | | 1.17 | |
| ko04120 | | Ubiquitin mediated proteolysis | | 3/47 | 55/2046 | | | 0.130231 | | 1.17 | |
| ko00524 | | Neomycin, kanamycin and gentamicin biosynthesis | | 1/47 | 4/2046 | | | 0.088832 | | 1.11 | |
| ko04140 | | Regulation of autophagy | | 2/47 | 14/2046 | | | 0.039436 | | 1.09 | |
| ko00400 | | Phenylalanine, tyrosine and tryptophan biosynthesis | | 2/47 | 20/2046 | | | 0.075501 | | 1.07 | |
|  | |  | |  | | |  |  | |  | |
|  | |  | |  | | |  |  | |  | |
|  | |  | |  | | |  |  | |  | |
|  | |  | |  | | |  |  | |  | |
|  | |  | |  | | |  |  | |  | |
|  | |  | |  | | |  |  | |  | |
|  | |  | |  | | |  |  | |  | |
|  | |  | |  | | |  |  | |  | |
|  | |  | |  | | |  |  | |  | |
|  | |  | |  | | |  |  | |  | |
|  | |  | |  | | |  |  | |  | |
|  | |  | |  | | |  |  | |  | |
|  | |  | |  | | |  |  | |  | |
|  | |  | |  | | |  |  | |  | |
|  | |  | |  | | |  |  | |  | |
|  | |  | |  | | |  |  | |  | |
|  | |  | |  | | |  |  | |  | |
|  | |  | |  | | |  |  | |  | |
|  | |  | |  | | |  |  | |  | |
|  | |  | |  | | |  |  | |  | |
|  | |  | |  | | |  |  | |  | |

**Table S8** Analysis of KEGG enrichment of CM4/CM3

| **ID** | | **Description** | | **Significant** | | **Annotated** | | **Pvalue** | | **log_2_FoldChange** | |
| --- | --- | --- | --- | --- | --- | --- | --- | --- | --- | --- | --- |
| ko01212 | | Fatty acid metabolism | | 10/199 | | 29/2046 | | 0.000232 | | 4.63 | |
| ko00520 | | Amino sugar and nucleotide sugar metabolism | | 14/199 | | 54/2046 | | 0.000414 | | 3.79 | |
| ko01200 | | Carbon metabolism | | 22/199 | | 108/2046 | | 0.000446 | | 3.67 | |
| ko00072 | | Synthesis and degradation of ketone bodies | | 3/199 | | 6/2046 | | 0.014526 | | 3.67 | |
| ko00100 | | Steroid biosynthesis | | 8/199 | | 24/2046 | | 0.001299 | | 3.39 | |
| ko00670 | | One carbon pool by folate | | 4/199 | | 12/2046 | | 0.022992 | | 3.37 | |
| ko00720 | | Carbon fixation pathways in prokaryotes | | 4/199 | | 12/2046 | | 0.022992 | | 3.37 | |
| ko00220 | | Arginine biosynthesis | | 7/199 | | 20/2046 | | 0.00191 | | 3.29 | |
| ko00960 | | Tropane, piperidine and pyridine alkaloid biosynthesis | | 4/199 | | 8/2046 | | 0.004442 | | 3.29 | |
| ko01230 | | Biosynthesis of amino acids | | 21/199 | | 122/2046 | | 0.00553 | | 3.29 | |
| ko01210 | | 2-Oxocarboxylic acid metabolism | | 8/199 | | 36/2046 | | 0.019227 | | 3.29 | |
| ko00630 | | Glyoxylate and dicarboxylate metabolism | | 10/199 | | 33/2046 | | 0.000757 | | 3.27 | |
| ko00020 | | Citrate cycle (TCA cycle) | | 9/199 | | 28/2046 | | 0.000869 | | 3.26 | |
| ko00640 | | Propanoate metabolism | | 6/199 | | 24/2046 | | 0.023723 | | 3.18 | |
| ko02020 | | Two-component system | | 6/199 | | 18/2046 | | 0.005366 | | 2.67 | |
| ko01040 | | Biosynthesis of unsaturated fatty acids | | 5/199 | | 14/2046 | | 0.007974 | | 2.63 | |
| ko04011 | | MAPK signaling pathway - yeast | | 14/199 | | 60/2046 | | 0.001301 | | 2.13 | |
| ko00531 | | Glycosaminoglycan degradation | | 3/199 | | 6/2046 | | 0.014526 | | 2.09 | |
| ko00604 | | Glycosphingolipid biosynthesis - ganglio series | | 2/199 | | 3/2046 | | 0.026436 | | 2.09 | |
| ko00260 | | Glycine, serine and threonine metabolism | | 11/199 | | 44/2046 | | 0.002399 | | 1.7 | |
| ko00514 | | Other types of O-glycan biosynthesis | | 3/199 | | 4/2046 | | 0.003369 | | 1.63 | |
|  | |  | |  | |  | |  | |  | |
|  | |  | |  | |  | |  | |  | |
|  | |  | |  | |  | |  | |  | |
|  | |  | |  | |  | |  | |  | |
|  | |  | |  | |  | |  | |  | |
|  | |  | |  | |  | |  | |  | |
|  | |  | |  | |  | |  | |  | |
|  | |  | |  | |  | |  | |  | |
|  | |  | |  | |  | |  | |  | |
|  | |  | |  | |  | |  | |  | |
|  | |  | |  | |  | |  | |  | |
|  | |  | |  | |  | |  | |  | |
|  | |  | |  | |  | |  | |  | |
|  | |  | |  | |  | |  | |  | |
|  | |  | |  | |  | |  | |  | |
|  | |  | |  | |  | |  | |  | |
|  | |  | |  | |  | |  | |  | |
|  | |  | |  | |  | |  | |  | |
|  | |  | |  | |  | |  | |  | |
|  | |  | |  | |  | |  | |  | |
|  | |  | |  | |  | |  | |  | |

**Table S9** Detection of carotenoid content in *C. militaris*

| **ID** | **OD445-1** | **OD445-2** | **OD445-3** | **Concentration 1 (μg/g)** | **Concentration 2 (μg/g)** | **Concentration 3 (μg/g)** | **Average concentration (μg/g)** | **Standard error** |
| --- | --- | --- | --- | --- | --- | --- | --- | --- |
| *CmUGT1-1* | 0.2489 | 0.2484 | 0.2481 | 233.34 | 232.87 | 232.59 | 232.94 | 0.38 |
| *CmUGT1-2* | 0.1741 | 0.1743 | 0.1737 | 163.22 | 163.41 | 162.84 | 163.16 | 0.29 |
| *CmUGT1-3* | 0.1196 | 0.119 | 0.1189 | 112.12 | 111.56 | 111.47 | 111.72 | 0.35 |
| WT-1 | 0.0569 | 0.0564 | 0.0564 | 53.34 | 52.87 | 52.87 | 53.03 | 0.27 |
| WT-2 | 0.0660 | 0.0671 | 0.0658 | 61.87 | 62.91 | 61.69 | 62.16 | 0.66 |
| WT-3 | 0.0744 | 0.0721 | 0.073 | 69.75 | 67.59 | 68.44 | 68.59 | 1.09 |
| WT-Average | 0.0658 | 0.0652 | 0.0651 | 53.03 | 62.16 | 68.59 | 61.26 | 7.82 |

**Table S10** Detection of polysaccharides content in *C. militaris*

| **ID** | **OD540**  **-1** | **OD540**  **-2** | **OD540**  **-3** | **Total sugar concentration1**  **(μg/mL)** | **Total sugar concentration 2**  **(μg/mL)** | **Total sugar concentration 3**  **(μg/mL)** | **Reducing sugar concentration 1**  **(μg/mL)** | **Reducing sugar concentration 2**  **(μg/mL)** | **Reducing sugar concentration 3**  **(μg/mL)** | **Polysaccharides concentration 1**  **(μg/mL)** | **Polysaccharides concentration 2**  **(μg/mL)** | **Polysacchari des concentration 3**  **(μg/mL)** | **Average concentration**  **(μg/mL)** | **Standard error** |  |  |
| --- | --- | --- | --- | --- | --- | --- | --- | --- | --- | --- | --- | --- | --- | --- | --- | --- |
| *CmUGT1-1* | 0.06 | 0.06 | 0.07 | 317.23 | 317.23 | 317.77 | 30.15 | 30.15 | 35.39 | 287.08 | 287.08 | 282.38 | 285.52 | 2.71 |  |  |
| *CmUGT1-2* | 0.19 | 0.19 | 0.19 | 330.82 | 331.36 | 330.27 | 96.73 | 96.20 | 96.73 | 234.09 | 235.15 | 233.54 | 234.26 | 0.82 |  |  |
| *CmUGT1-3* | 0.02 | 0.02 | 0.02 | 62.14 | 62.47 | 62.36 | 8.65 | 8.13 | 9.17 | 53.49 | 54.34 | 53.18 | 53.67 | 0.60 |  |  |
| WT-1 | 0.01 | 0.01 | 0.01 | 89.16 | 89.16 | 88.31 | 5.33 | 5.11 | 5.33 | 83.83 | 84.05 | 82.98 | 83.62 | 0.56 |  |  |
| WT-2 | 0.01 | 0.01 | 0.01 | 89.16 | 89.16 | 89.34 | 5.33 | 5.31 | 5.32 | 83.83 | 83.85 | 84.02 | 83.9 | 0.10 |  |  |
| WT-3 | 0.01 | 0.02 | 0.01 | 89.16 | 105.16 | 88.31 | 5.33 | 7.61 | 5.33 | 83.83 | 97.55 | 82.98 | 88.12 | 8.18 |  |  |
| WT-Average | 0.01 | 0.01 | 0.01 | 89.16 | 89.16 | 88.31 | 5.33 | 5.11 | 5.33 | 83.83 | 84.05 | 82.98 | 83.62 | 0.56 |  |  |

**Table S11** Cordycepin content in supernatant of *C. militaris*

| **ID** | **Repeat 1**  **(μV/s)** | **Repeat 2**  **(μV/s)** | **Repeat 3**  **(μV/s)** | **Concentration 1**  **(mg/mL)** | **Concentration 2**  **(mg/mL)** | **Concentration 3**  **(mg/mL)** | **Average concentration**  **(mg/mL)** | **Average volume**  **(mL)** | **mg/L** | **Standard error** |
| --- | --- | --- | --- | --- | --- | --- | --- | --- | --- | --- |
| *CmUGT1-1* | 3734041 | 3777715 | 3731384 | 0.073 | 0.074 | 0.073 | 0.073 | 65 | 47.45 | 0.52 |
| *CmUGT1-2* | 3614639 | 3700473 | 3732906 | 0.071 | 0.073 | 0.073 | 0.072 | 84 | 60.84 | 1.22 |
| *CmUGT1-3* | 6529480 | 6588429 | 6630984 | 0.129 | 0.130 | 0.131 | 0.130 | 75 | 97.50 | 1.02 |
| WT-1 | 1419898 | 1119898 | 1319898 | 0.029 | 0.023 | 0.027 | 0.027 | 86 | 23.22 | 3.05 |
| WT-2 | 1649900 | 1549900 | 1349900 | 0.034 | 0.032 | 0.028 | 0.031 | 80 | 24.80 | 3.05 |
| WT-3 | 1019304 | 919304 | 951930 | 0.021 | 0.019 | 0.020 | 0.020 | 91 | 18.20 | 1.02 |
| WT-Average | 1363034 | 1196367 | 1207243 | 0.028 | 0.025 | 0.025 | 0.026 | 86 | 22.36 | 1.86 |

**Table S12** Cordycepin content in mycelium of *C. militaris*

| **ID** | **Repeat 1**  **(μV/s)** | **Repeat 2**  **(μV/s)** | **Repeat 3**  **(μV/s)** | **Concentration 1**  **(μg/g)** | **Concentration 2**  **(μg/g)** | **Concentration 3**  **(μg/g)** | **Average concentration**  **(μg/g)** | **Average dry weight**  **(g)** | **Standard error** |
| --- | --- | --- | --- | --- | --- | --- | --- | --- | --- |
| *CmUGT1-1* | 369382 | 365123 | 352157 | 658.764 | 650.246 | 624.314 | 644.441 | 3.082 | 17.94 |
| *CmUGT1-2* | 573528 | 563725 | 554528 | 1067.056 | 1047.45 | 1029.056 | 1047.854 | 2.890 | 19.00 |
| *CmUGT1-3* | 392585 | 432573 | 452530 | 705.170 | 785.146 | 825.060 | 771.792 | 1.950 | 61.05 |
| WT-1 | 103541 | 99562 | 90423 | 127.082 | 119.124 | 100.846 | 115.684 | 2.030 | 13.45 |
| WT-2 | 99374 | 123658 | 104286 | 118.748 | 167.316 | 128.572 | 138.212 | 1.930 | 25.67 |
| WT-3 | 120236 | 114268 | 98362 | 160.472 | 148.536 | 116.724 | 141.911 | 2.350 | 22.61 |
| WT-Average | 107717 | 112496 | 97690 | 135.434 | 144.992 | 115.381 | 131.936 | 2.100 | 15.11 |
